# Simple and Versatile 3D Printed Microfluidics Using Fused Filament Fabrication

**DOI:** 10.1371/journal.pone.0152023

**Published:** 2016-04-06

**Authors:** Alex J. L. Morgan, Lorena Hidalgo San Jose, William D. Jamieson, Jennifer M. Wymant, Bing Song, Phil Stephens, David A. Barrow, Oliver K. Castell

**Affiliations:** 1 Cardiff School of Engineering, Cardiff University, Queen’s Building, The Parade, Cardiff, CF24 3AA, United Kingdom; 2 School of Pharmacy and Pharmaceutical Sciences, Cardiff University, Redwood Building, King Edward VII Ave, Cardiff, CF10 3NB, United Kingdom; 3 Oral and Biomedical Sciences, Cardiff Institute of Tissue Engineering and Repair, School of Dentistry, Cardiff University, Heath Park, Cardiff, CF14 4XY, United Kingdom; Chang Gung University, TAIWAN

## Abstract

The uptake of microfluidics by the wider scientific community has been limited by the fabrication barrier created by the skills and equipment required for the production of traditional microfluidic devices. Here we present simple 3D printed microfluidic devices using an inexpensive and readily accessible printer with commercially available printer materials. We demonstrate that previously reported limitations of transparency and fidelity have been overcome, whilst devices capable of operating at pressures in excess of 2000 kPa illustrate that leakage issues have also been resolved. The utility of the 3D printed microfluidic devices is illustrated by encapsulating dental pulp stem cells within alginate droplets; cell viability assays show the vast majority of cells remain live, and device transparency is sufficient for single cell imaging. The accessibility of these devices is further enhanced through fabrication of integrated ports and by the introduction of a Lego^®^-like modular system facilitating rapid prototyping whilst offering the potential for novices to build microfluidic systems from a database of microfluidic components.

## Introduction

For many years microfluidics has been hailed as a field that could transform the way research is performed in the biological and chemical communities [1–3]. Microfluidics enables the precise manipulation of fluids on the small scale, enabling the use of small sample volumes, reduced costs, increased throughput, and parallel and sequential processing easily amenable to automation and portability on a scale beyond that achievable by manual or traditional robotic manipulation [4]. Microfluidics exploits the unique behaviour of fluids on the micro-scale where surface and viscous forces dominate over gravity and inertia, giving rise to laminar flow in single phase systems, and reproducible and programmable droplet flow in multiphase systems [5]. These advantageous fluid dynamics have been used in diverse fields with applications in cell encapsulation [6], DNA analysis [7, 8], drug prototyping [9], high throughput screening [10, 11], cell and droplet sorting and separation [12–15], chemical synthesis [16], chemical separations [17] radiopharmaceutical production [18], proteomics [19] and diagnostic technologies [20] amongst others.

Despite these unique properties and diverse application areas, microfluidics has largely remained a somewhat specialist research area with limited uptake by those who could benefit most from the technology. Two key factors have limited the take-up by wider disciplines; manufacturing and versatility. Firstly, traditional microfluidic manufacturing methods, such as soft lithography, require skills and equipment that is often not readily available in a typical biology, chemistry or pharmacy laboratory [21, 22]. Secondly, the fixed nature of the fabricated devices limits iterative process optimisation or flexible application.

### Overcoming the fabrication barrier

The barriers to wider uptake are clear if the fabrication technique of soft lithography with polydimethylsiloxane (PDMS) is taken as just one example. Despite the advantages to this fabrication technique, the creation of a PDMS device requires knowledge of- and access to- photolithographic and plasma bonding equipment. Similar know-how and equipment requirements are true of other common fabrication techniques such as laser fabrication, micro milling, ion-etching and photolithographic fabrication. Berthier *et al* summarise the problem stating “adoption of microscale technologies by biologists hinges on… successful collaborations between engineers and biologists… and the establishment of standard platforms that… are widely accessible and available to the biology community at large”[23]. Here we present a microfluidic platform capable of addressing this challenge, taking versatile microfluidics into the laboratories of the wider scientific community at low cost and without the need for specialist equipment or expertise.

Recently, extrusion based 3D printing (fused filament fabrication (FFF)) has developed into a readily available consumer technology. As ease of use and resolutions have improved, and prices fallen, the ability to create custom objects quickly and easily has become available to all, causing a paradigm shift in small scale manufacturing. The ease of creating custom objects and devices cheaply and easily is democratising previously specialist areas of manufacturing, greatly increasing the capability of non-experts, and allowing cheap and rapid prototyping and production.

Until recently, 3D printing has been limited by resolution or cost of the printers [24, 25]. However, recent advances mean that microfabrication is now possible off-the-shelf, without sophisticated manufacturing centres [26] and advances continue apace meaning the possibilities are likely to increase further still. 3D printing has been applied to micro- and millifluidics in combination with other techniques [27] or as a fabrication process in its own right [22, 24, 26, 28–31]. Stereolithography (SL) printing, which has been more commonly used for microfluidics, involves the layer-by-layer photocuring of a polymer resin enabling the manufacture of three-dimensional objects from a reservoir of liquid resin. The more prevalent and accessible printing technology, fused filament fabrication (FFF), has often been discounted for the production of microfluidic devices [22]. This type of printing has often been dismissed due to leaking between extruded layers, along with the lack of transparency and a perceived lack of resolution and accuracy [32]. FFF printing (also known as fused deposition modelling (FDM) or extrusion printing) is an additive manufacturing process where the extrusion of molten polymer layer-by-layer enables the construction of 3D objects. By this method, proof-of-concept 800μm diameter microfluidic channels have been produced in polypropylene [24]. Whilst SL printing can provide greater channel resolution than FFF, it has rarely been demonstrated with channel dimensions below 500μm. Significantly, the increased complexity and cost of the SL printing process means that fabrication of such devices has often been outsourced to specialists [22, 29, 33, 34] creating comparable barriers to uptake as with traditional manufacturing methods. With printers currently available at prices below £1000, FFF offers much higher levels of accessibility meaning most laboratories should be capable of fabricating their own devices in-house, facilitating cheap production and fast prototyping for a wide range of applications.

### Design Concept

**Fig 1:**
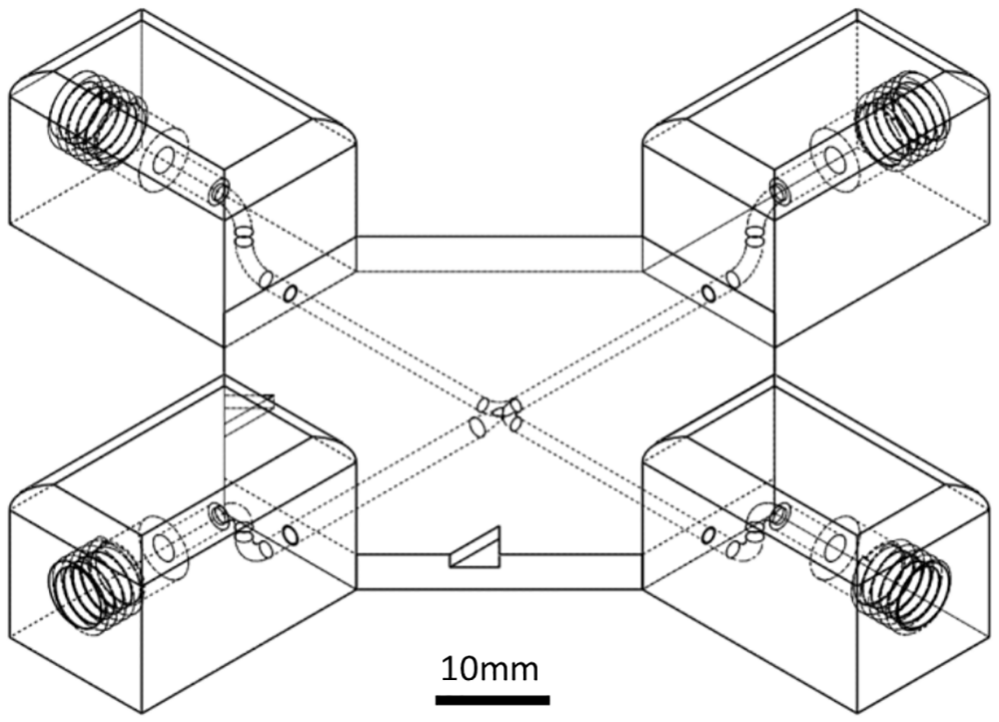
Schematic illustration of a flow focusing junction showing the incorporation of finger-tight fitting ports. Once this model has been created using SOLIDWORKS, or similar design software, the device can be created on a 3D printer with a simple press of a button.

The 3D printed devices presented here were designed to allow easy integration with traditional fluid handling systems. As such, threaded ports at the inlets and outlets allow for the use of simple PEEK finger-tight fittings to connect devices to tubing and pumps. An example design is shown in Fig 1. For further accessibility connectable fluidic modules were also designed. The fluidic modules were based on the Lego^®^ blocks that are familiar around the world and can be simply clipped together creating leak-free re-configurable microfluidic systems.

## Experimental

### Device Fabrication

Devices were produced via fused filament fabrication using an Ultimaker 2 printer. Devices were printed at a print speed of 30mm/s at a nozzle temperature of 215°C. The modules were printed with 100% fill density on a build plate that was heated to 70°C. Device schematics were designed in Solidworks 2013 before being converted to a print pattern using Simplify3D 2.2.2 software. Alternating 50μm thick layers were printed such that the pattern ran perpendicular and then parallel to the length of the device, enabling leak-free and transparent devices to be printed with clear Polylactic acid (PLA). 3D printed microfluidic devices were also produced stereolithographically using a Miicraft printer with 100μm layers and 25s curing time. Full print settings can be found in S1 Table.

### Pressure Test

To characterise the fluidic interfacing and connections between modules, the maximum pressure that the connections could withstand before leaking was measured. This was achieved by blocking the outlet of one module while pumping water into the inlet of a connected module at a constant flow rate of 12ml/hr (Rheos 2000, Flux Instruments). The pressure required to drive the water was monitored throughout the experiment. Leaks were detected by visual inspection. Pressure data was recorded using the pump control software (Janeiro II 2.6).

### Materials and Devices

Initial tests were performed to investigate the suitability of a number of commercially available printer filaments. T-junction devices for droplet generation were created with 1mm diameter channels and the output of the device was observed. Sunflower oil and Water (containing Red Silverspoon dye) were delivered via syringe pumps (Legato 210, KD Scientific) to create water in oil droplet emulsions. Devices were fabricated using PLA filament (Ultimaker PLA 2.85mm), polyethylene terephthalate (PET) transparent filament (Taulman t-glase 3mm), a modified acrylonitrile butadiene styrene (ABS) transparent filament (Bendlay 3mm), and transparent PLA (Faberdashery, Crystal Clear 3mm). SL printed devices were produced using DETAX Luxaprint clear resin.

Flow-focusing junctions were tested using mineral oil (Sigma-Aldrich) dyed with Oil Blue N (Sigma-Aldrich). Further tests used an alginate solution that contained 2% alginate in distilled water with 7.5mg/ml of calcium carbonate, CaCO_3_, (Sigma-Aldrich) (with SilverSpoon red food colouring for visualisation). The solution was stirred magnetically for 1h at 50°C to dissolve the alginate. To create oil in water emulsions, a 10mM aqueous oleic acid (Sigma-Aldrich) solution at pH13 was used with pH adjustment achieved with sodium hydroxide (Sigma-Aldrich) in deionised water (4g/L). The solution was then sonicated for 1 minute after the oleic acid was added to ensure that the solution was monophasic. Measurements of the droplets created on the flow-focusing junctions were taken using a high speed camera (Megaspeed) in combination with NIS Elements 3.2.

### Fidelity Measurements

To assess the quality and accuracy of the 3D printing, a series of channels were printed with diameters ranging from 400μm-1.5mm in increments of 100μm. The channel dimensions were then measured using a Nikon AZ100 microscope with NIS Elements 3.2 software. The channels were printed using both FFF printing and SL printing. FFF printed channels were printed in both horizontal and vertical orientations. Horizontally printed channels were measured in two directions to assess both the width and height of the channels. 25μm, 50μm and 100μm layer thickness prints were assessed. The measured channel dimensions were compared to the original dimensions specified in the models created in Solidworks.

### Stem Cell Encapsulation

Human dental pulp stem cells (hDPSCs) were encapsulated in alginate capsules (1x10^6^ cells/mL). The cells were obtained from third molars (donors aged 17–20) with all patient’s informed written consent in accordance with the Research and Human Tissue Act 2004. Ethical approval was granted by South East Wales Research Ethics Committee of the National Research Ethics Service (permission number: 07/WESE04/84. Ethical documentation can be found in S1–S3 Figs) and cultured in α-modification minimum essential medium (αMEM) containing 2mM glutamine, ribonucleosides and deoxyribonucleosides (Life Technologies, UK). The medium was supplemented with 1% (v/v) penicillin/streptomycin, 10% (v/v) heat-inactivated foetal bovine serum (FBS) (Life Technologies, UK) and 100μM l-ascorbic acid 2-phosphate (Sigma-Aldrich, UK). The medium was changed every 2–3 days until cells reached 80–90% confluence. Upon reaching confluence, culture medium was removed by aspiration and the cells washed with phosphate buffered saline (PBS) (Sigma-Aldrich, UK). Cells were dissociated by adding trypsin-EDTA 0.25% (v/v) (Sigma-Aldrich, UK) and returned to the incubator for 3–5 minutes until they became rounded and detached. The trypsin was neutralised by adding culture medium. The medium and cell solution were then transferred to 15ml falcon tubes and centrifuged at 1500rpm for 5 minutes. After discarding the supernatant, pellets were resuspended in medium and cell counts performed using a haemocytometer. Finally, cells were centrifuged again and resuspended at a density of 1 million cells per ml of alginate in 2% low viscosity alginate solution (AO682, Sigma Aldrich, UK). The alginate solution was created by adding 5mg/ml of calcium carbonate (Sigma-Aldrich) to αMEM supplemented with 1% (v/v) penicillin/streptomycin before adding 20mg/ml of alginate and stirring for 2 hours at 50°C. Monodisperse alginate droplets containing stem cells were created on the 3D printed devices within sunflower oil continuous phase. Alginate droplets were gelled for approximately 10 minutes on exiting the chip in a bath solution of sunflower oil containing glacial acetic acid (0.3% v/v). After gelling the capsules were washed in culture medium and, after varying periods of time in culture, viability assessed using a LIVE/DEAD^®^ Viability/Cytotoxicity assay kit for mammalian cells (Invitrogen), with calcein-AM (green) indicating intracellular esterase activity in live cells, and ethidium homodimer-1 (red) fluorescence indicating loss of plasma membrane integrity in dead cells. Laser scanning confocal imaging of encapsulated cells was performed using a Leica SP5 Confocal Microscope and LAS AF imaging software (Leica Microsystems, Germany). Images of encapsulated cells were acquired from confocal Z scan over a depth of 600 μm.

### Plasma membrane labelling of stem cells

Cells were centrifuged at 400g for 5 min. The supernatant was discarded and cells were resuspended in 1 mL of sterile PBS containing CellMask Orange (C10045, Molecular Probes) at a concentration of 5 ng/mL. The cells were incubated with this membrane stain for 10 min at room temperature before being centrifuged at 900g for 2 min. The supernatant was discarded and the cell pellet was washed by resuspension in sterile PBS and incubation at room temperature for 1 min. The cells were then recentrifuged (900g, 2 min) and resuspended once more in 1 mL of PBS for downstream analysis. Droplets containing sulforhodamine b (250 nM and 25 nM) and aqueous suspension of CellMask Orange labelled stem cells were imaged under flow conditions in the transparent PLA device equivalent to those used in the device transparency tests. Imaging was conducted at 4x or 10x magnification with 532 nm illumination via a TIRF fibre couple interfaced in epi-fluorescence mode on a Nikon Eclipse Ti-U inverted microscope. A 590/50 nm fluorescence emission filter (Chroma, USA) filtered emitted light prior to imaging on an Andor iXon camera at an acquisition frame time of 18 ms.

## Results & Discussion

**Fig 2:**
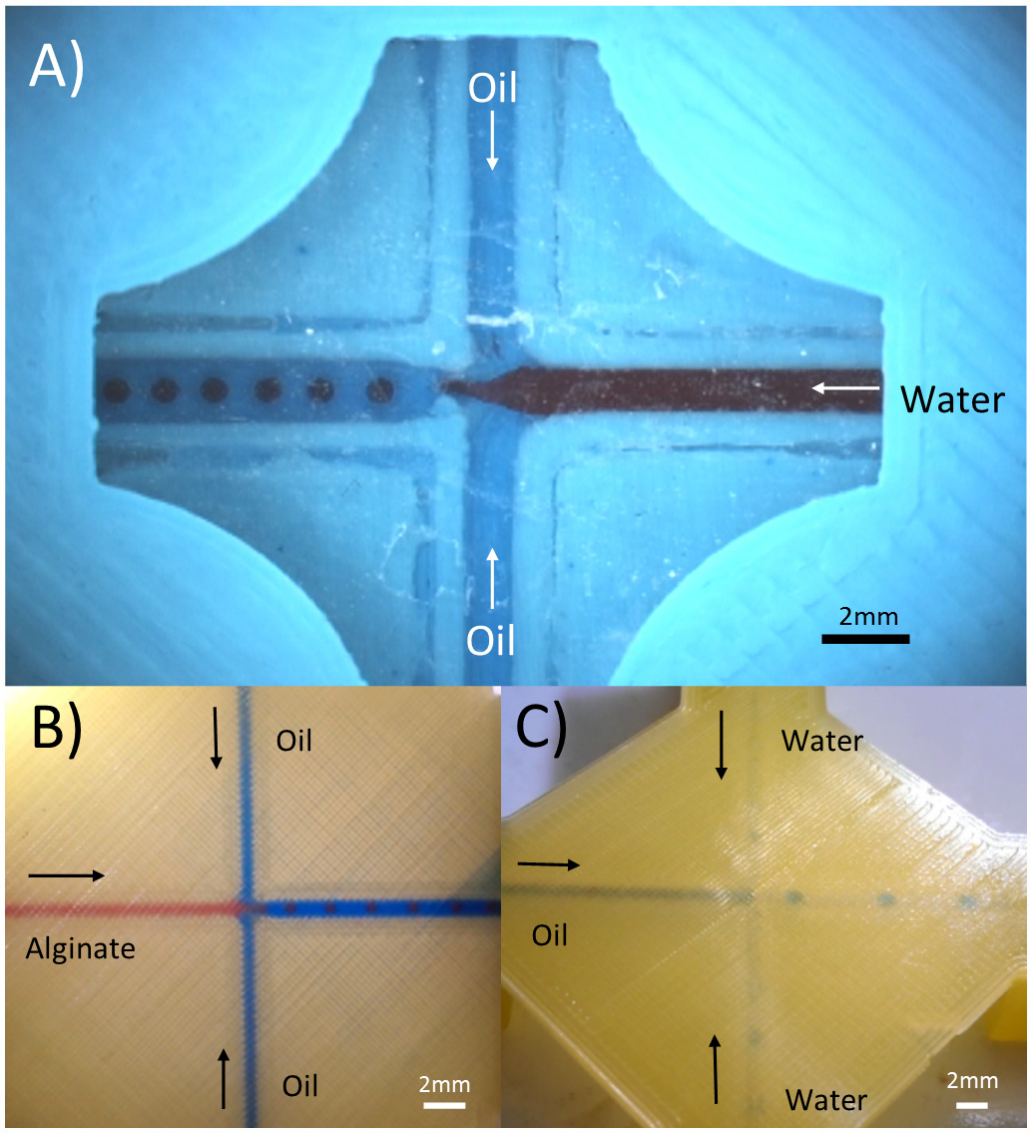
Extrusion 3D printed flow focusing junctions. A) Water in oil droplets formed in a PLA module that has a glass observation window embedded within it. B) Alginate solution droplets in sunflower oil generated in a semi-transparent PLA module. Oil flow rate: 3ml/hr, alginate flow rate: 1ml/hr C) Mineral oil droplets in water and 10mM oleic acid carrier phase. Generated on a semi transparent PLA device. Water flow rate: 4ml/hr, oil flow rate: 1.5ml/hr. Junctions A and C have inlet channel widths of 1mm with a 1.4mm wide outlet. Junction B has 600μm wide channels with a 900μm wide outlet.

To demonstrate the capabilities of FFF printed microfluidic devices, flow focusing junctions were created to form droplets in a controlled manner (Fig 2). Droplets of water in oil are readily formed. In addition, alginate droplets in oil, and oil droplets in a continuous phase of water and oleic acid were also demonstrated, illustrating the feasibility of consistent droplet formation in 3D printed microfluidic devices. Importantly, Fig 2 also shows that it is possible to observe the fluid flow within 3D printed PLA devices, either through the use of an embedded glass observation window for microscopy applications (Fig 2A), or through the use of semi-transparent PLA with sufficiently thin walls (Fig 2B and 2C).

**Fig 3:**
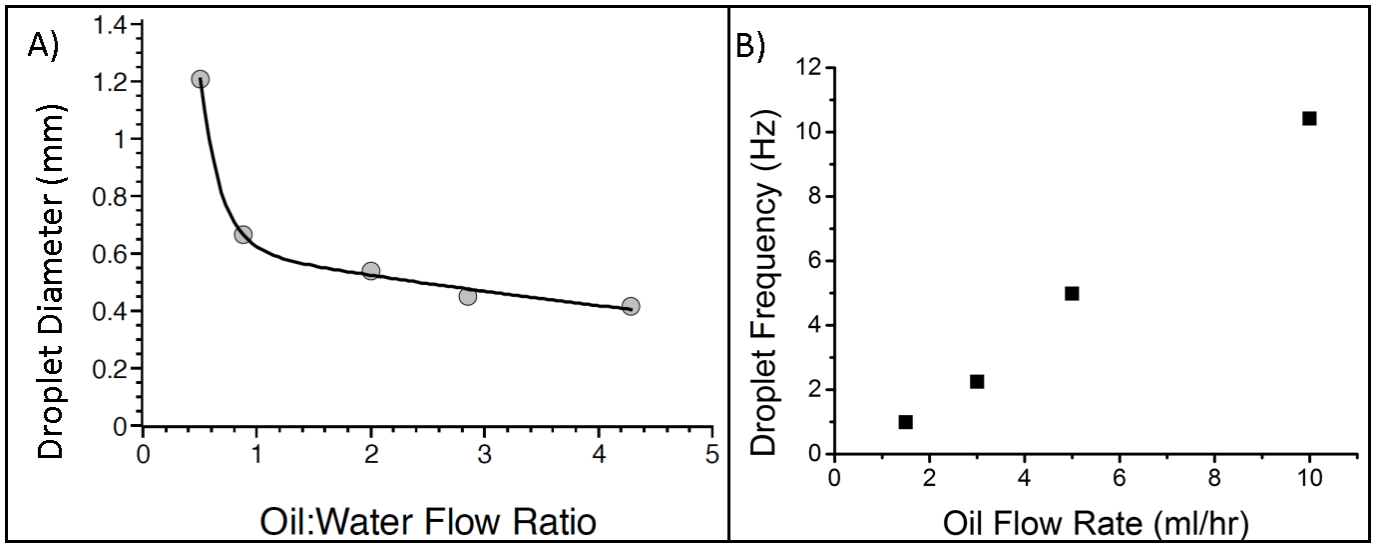
Droplet data from a 3D printed flow-focusing junction (Fig 2A). Water droplets formed in mineral oil. A) Droplet diameter as the flow rate into each oil inlet is increased. The oil flow rate was increased as multiples of the water flow rate. Curve added to illustrate the trend. The error ranged from 1.3–3.3% based on 95% confidence level. B) Droplet frequency as the oil and water flow rates into each inlet are increased (fixed oil:water flow rate ratio of 2:1). For example, at the 10ml/hr data point both oil inlets are set to 10ml/hr whilst the water inlet is set to 5ml/hr. The error the error for the 1.5ml/hr data pointwas 13% due to data gathering using an alternative camera with a lower frame rate, the error for all the other data points ranged from 0.2–1.2% based on 95% confidence. In both A) and B) 10 droplets were measured for each data point (n = 10). Error bars not plotted as they are obscured by data points.

The PLA flow-focusing junction with glass observation window (Fig 2A) was used to assess the reproducibility and control of water in oil droplet formation (Fig 3). Droplet size can be controlled by varying the ratio of the water and oil flow rates with a higher oil flow rate, relative to the water flow rate, leading to smaller droplets. The channel geometry and fluid properties define the minimum droplet size. The droplet generation frequency was investigated by increasing the total flow rate at a fixed oil:water flow ratio. This results in an increase in droplet frequency without altering the diameter. Droplets of a consistent diameter of 504μm (±18μm) were produced throughout the frequency test, as droplet production frequency increased from 1 to 10.4Hz. Frequency variation was very small as evidenced by the small fluctuation range of 0.2–1.2% (95% confidence interval), indicative of high stability operation. However, at the lowest flow rate of (1.5 ml/hr) 13% variation in production frequency was measured, this is due to this data being collected at a lower frame rate using a different camera than the other data points. The behaviour of this 3D printed flow focusing junction is consistent with previous studies of flow focusing junctions [35].

### Fidelity Tests

**Fig 4:**
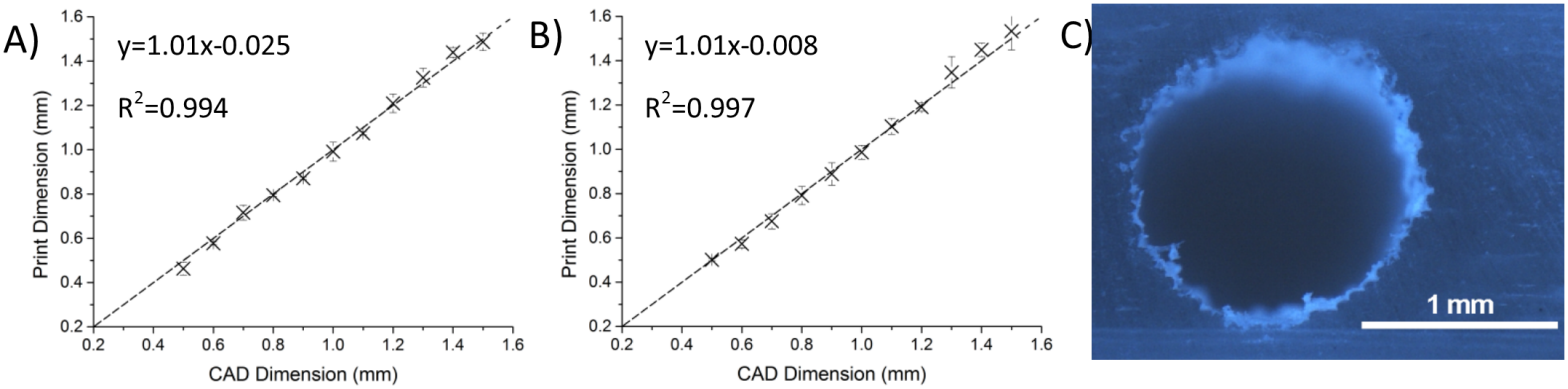
3D printer fidelity measurements. a and b) Comparisons of measured and desired dimensions of 3d printed circular channels. Dotted line indicates perfect fidelity between the CAD model and the printed channel. a) Measured width of a horizontally printed channel using FFF printing with 50μm layers. b) Measured depth of a horizontally printed channel using FFF printing with 50μm layers. c) Image of a 1.4mm diameter channels fabricated using extrusion 3D printing. 3 measurements were taken for each data point. Error bars indicate 95% confidence level.

A criticism of FFF printing has been a lack of fidelity between the printed dimensions and the original CAD model [32]. Here, we investigate the correlation between measured circular cross-section of printed channels compared to the originally designed geometry, under a range of print conditions (50μm layers shown in Fig 4 Further printer fidelity analysis can be found in S4 Fig, showing different layer thicknesses and a comparison with SL printing.). High accuracy is possible with the right print parameters (in this case 50μm layer height) with both the height and width of the channel within 2.5% of the designed channel dimensions on average. Fig 4 illustrates the consistency of dimensions in all directions, although there is some unavoidable roughness resulting from the layer by layer nature of FFF printing as can be seen in Fig 4C. Although surface roughness was not considered for optimisation in this work, typical surface roughness was characterised by interferometeric measurement (S5 Fig) of gold sputter coated devices fabricated at 1200 mm min^-1^, with 50 μm layer deposition, as employed in the transparency tests. Over a 1.4 x 1.4 mm area material surfaces were found to have peak-to-peak variation of 16.81 μm. The nature of the deposition method leads to peaks at the interface between two extruded ‘strips.’ Further optimisation of the surface roughness may be achievable, either through further optimisation of print parameters or post fabrication solvent treatment, but was deemed beyond the scope of this study and unnecessary given the successful functionality of the printed devices. As printing resolution improves, and fabrication dimensions reduce, surface roughness optimisation may become of greater significance.

The fidelity measurements demonstrate that the minimum channel dimension achievable was 500μm. Whilst this is relatively large, it is suitable for many microfluidic applications. Currently, this is defined by the size of current printer nozzles. However, with smaller nozzles that have been released recently and advancements in printer technology anticipated, this limit should come down, further widening the application areas for extrusion printed microfluidics as channel dimensions reduce.

### Device Transparency

**Fig 5:**
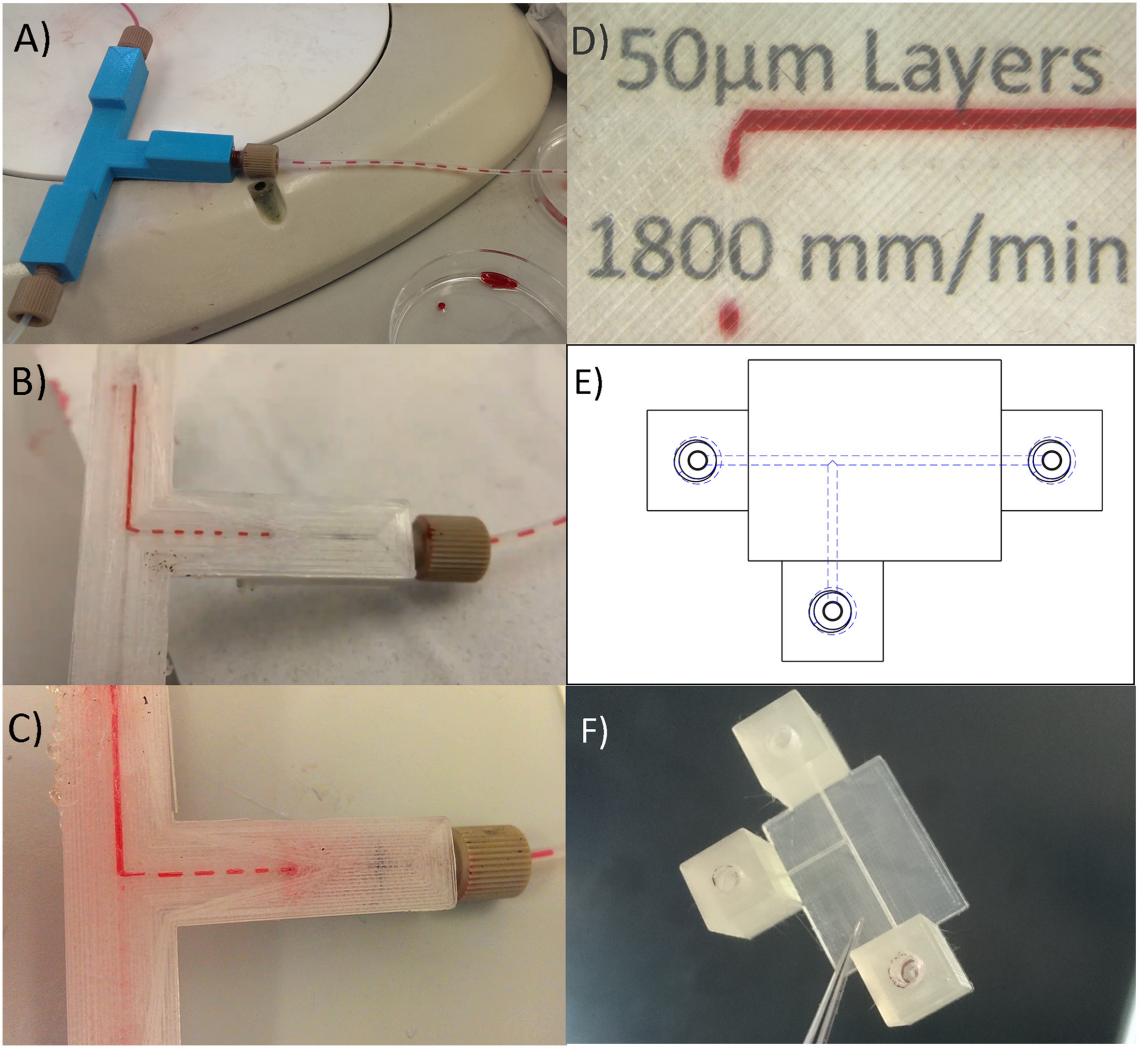
Overcoming the transparency barrier. A) A PLA T-junction using common coloured PLA. The device appears to be working effectively but only the fluids entering and exiting the device can be observed. Segmented flow is created by using water (red) and oil (clear). B) A device created using PET filament. Transparency is achieved using sufficiently thin walls, however wetting properties of the material result in unreliable droplet generation. This problem was also manifest with C) modified ABS (T-glase), likely due to permeation of the aqueous phase into the bulk material of the device, evidenced by the slight pink hue of the device in image C. D) Completely transparent microfluidic devices were achieved using transparent PLA with optimisation of print parameters. Printed text on paper placed under the device can easily be read through the printed material whilst the device is in operation. E) Schematic of the device shown in image D. F) Photograph of the device used in image D.

Although not essential for operation, it is often desirable to produce transparent microfluidic devices for observation or analytical purposes. Previously published FFF printed microfluidic devices have largely been opaque, although semi-transparent devices have also been achieved recently through the use of very thin side walls [36]. An example of this problem can be seen in Fig 5A (PLA T-junction). The inability to visualise the channel architecture or fluid within makes device development and operation more challenging. A number of off-the-shelf filaments were investigated together with optimisation of print conditions with the aim of improving transparency beyond that achieved using the semi-transparent PLA (Fig 2B and 2C).

**Fig 6:**
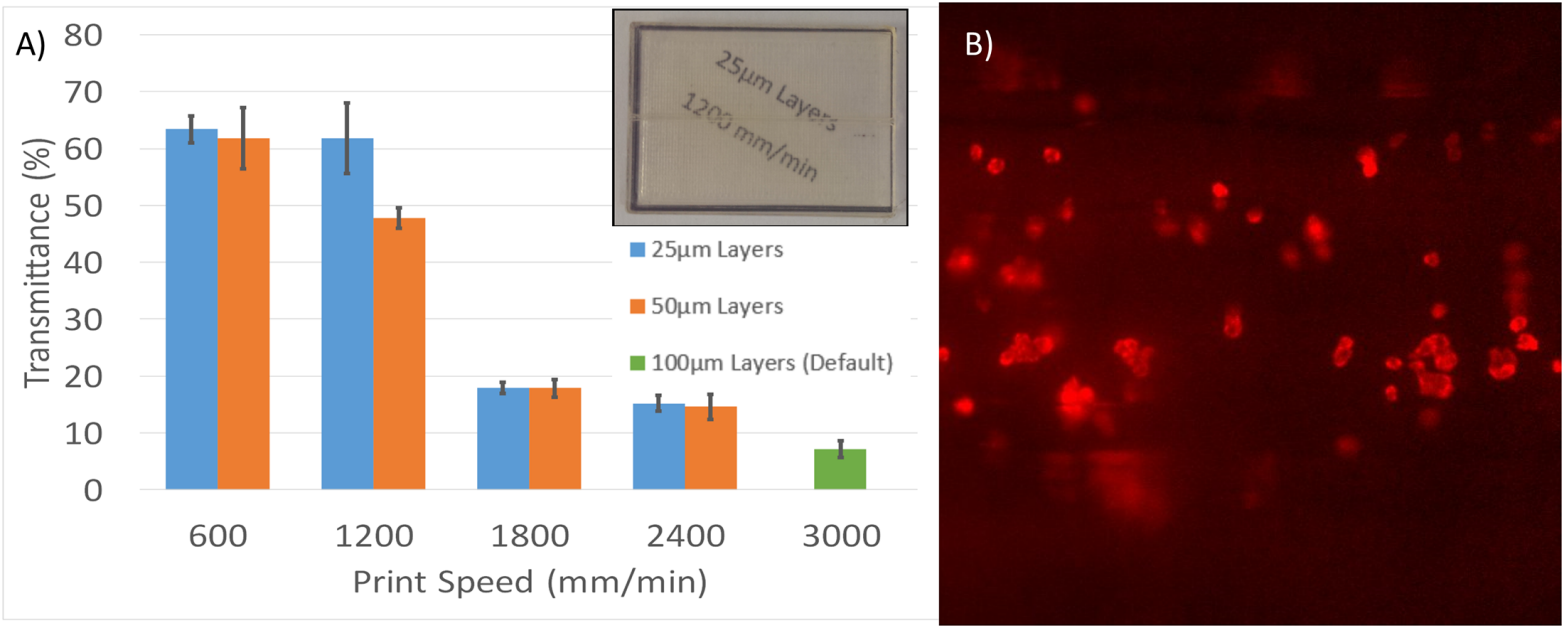
Optical transmission of 3D printed devices at various print speeds and layer heights. A) Fabricated device transmision as a percentage of the raw material at 550 nm. Comparing 25μm layers with 50μm layers, along with a comparison to the default Cura settings (with just the infill percentage changed to 100%) Inset: A 3D printed microfluidic device printed at 1200mm/min with 25μm layers, text printed on paper beneath the device detailing the manufacturing conditions is clearly legible through the device. A 1mm diameter channel runs through the centre of the device. B) individual stem cells labelled with membrane stain (CellMask Orange) imaged under flow conditions in a 3D printed transparent microfluidic device printed at 1200mm/min with 25μm layers. Device thickness 3 mm with the channel embedded at a depth of 1.5 mm. Videos of these cells in motion can be found online in S1–S3 Videos).

Both PET (Fig 5B) and ABS (Fig 5C) filaments were found to offer reasonable transparency through the use of thin channel walls enabling easy visualisation. However, the wetting characteristics of the material were found to be non-ideal for reliable droplet generation (See S6 Fig). Using transparent PLA together with the optimisation of print parameters of layer thickness, print speed and fill patterning, it was possible to achieve previously unparalleled levels of device transparency, eliminating the need to observe from one particular side and the need to use very thin walls. Sufficient transparency of print was achieved to readily visualise printed text through the device (Fig 5D) and for dynamic fluorescence imaging of single cells under flow conditions within channels (Fig 6B). To our knowledge this represents the first truly transparent FFF 3D-printed microfluidic device.

Optimisation of print parameters to achieve transparency were investigated and transparency quantified by transmission of visible light through the printed device compared to pristine polymer of equivalent transmission path length (Fig 6). Both fabrication layer height and print speed were found to have dramatic impact on transparency, with slower speeds and thinner layers improving transmission (Full transmission data within the visible light spectrum can be found in S7 Fig). An upper threshold appears to exist, above which decreasing speed does not improve the transmission. This limit is below that of non-printed, but moulded, PLA sheet of the same thickness. Scattering from the rougher printed surface may account for this. The transmission properties of the PLA filament material defines the ultimate transparency limit. Future development of print materials could improve this further. Whilst the transparency is currently limited to approximately 63% of that of the raw polymer, it is evident from Figs 5D and 5F and 6B that optical observation within these 3D printed devices is readily achievable. Droplets containing 25 nM sulforhodamine b and single stem cells stained with CellMask orange plasma membrane stain are readily visualised by fluorescence microscopy within the device channels. This represents a significant improvement in FFF 3D printed microfluidics. For applications where higher levels of optical transparency are desirable, we have demonstrated the possibility to incorporate a glass viewing window, as shown in Fig 2A.

Previous work has attempted to improve transparency through the use of thin walls, reducing the number of layers between the channel and the outside environment [36]. However, our findings here suggest that the key to achieving transparency in 3D printed devices is actually ensuring that there is no under extrusion. Indeed 25μm layer devices were found to offer better transmission than 50μm layer devices, despite having twice as many layers and print interfaces. Similarly, slower print speeds can also afford similar improvements as both configurations allow the same volume of material to be extruded over a longer period of time. Under extrusion results in the formation of cavities between layers of printed material leading to increased light scattering and consequently reduced transparency. The avoidance of under extrusion also significantly improves the seal of the microfluidic channels by eliminating the possibility of inter-layer gaps through which fluids may leak; a problem previously identified by Walbaur *et al*[37] as a possible limitation of 3D printing microfluidic devices. Although 25μm layers appear to offer improved transparency, the improved fidelity (Fig 4), afforded by 50μm layers makes this likely more suitable for producing microfluidic devices where precise control of channel geometry is important. It should be noted that fluids could be observed travelling through the device even with the least transparent print configuration in this experiment (50μm layers at 2400 mm/min), although this was highly limited with the default slicer settings.

### Stem Cell Encapsulation

**Fig 7:**
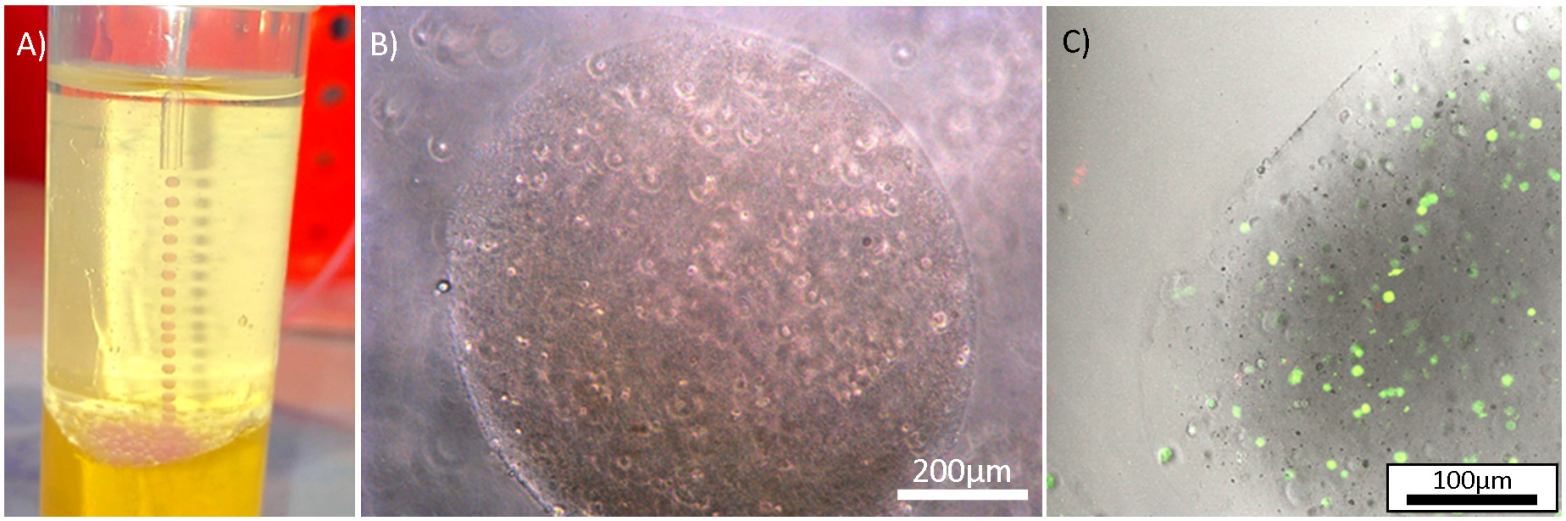
Live dental pulp stem cells encapsulated within alginate droplets created on a 3D printed device. A) Monodisperse alginate capsules exiting the microfluidic device B) Image of an alginate capsule containing stem cells. C) Confocal z-projection showing live encapsulated stem cells stained green and dead cells stained red. The image shows the edge of a capsule.

To demonstrate the practical utility of FFF 3D printed microfluidics for bio- and chemical applications, we created a device for the production of monodisperse alginate microspheres for the encapsulation of live stem cells. Such hydrogel cell encapsulation systems enable the three-dimensional culturing of cells and their easy manipulation [38]. In addition, cell encapsulation may provide a means for prolonged bio-therapeutic release in vivo [39]. The physical barrier provided by the encapsulating microsphere means that encapsulated stem cell systems are generating a large amount of interest for their potential application in tissue engineering and regenerative medicine therapies [40–42]. Monodisperse stem cell containing alginate droplets and calcium carbonate were created on the 3D printed devices within a sunflower oil continuous phase. Alginate droplets (800μm diameter) were gelled on entry into an acidified oil chamber. Partitioning of acid causes a reduction in pH within the alginate and liberation of Ca^2+^ from the calcium carbonate, giving rise to subsequent alginate gelation. After gelling the capsules were cleaned in culture medium and then cell viability assessed using a live/dead cell viability assay, with calcein-AM (green) indicating intracellular esterase activity within live cells, and ethidium homodimer-1 (red) fluorescence indicating loss of plasma membrane integrity in dead cells. Confocal imaging revealed that the vast majority of encapsulated cells remained live (Fig 7C).

### Modular System

**Fig 8:**
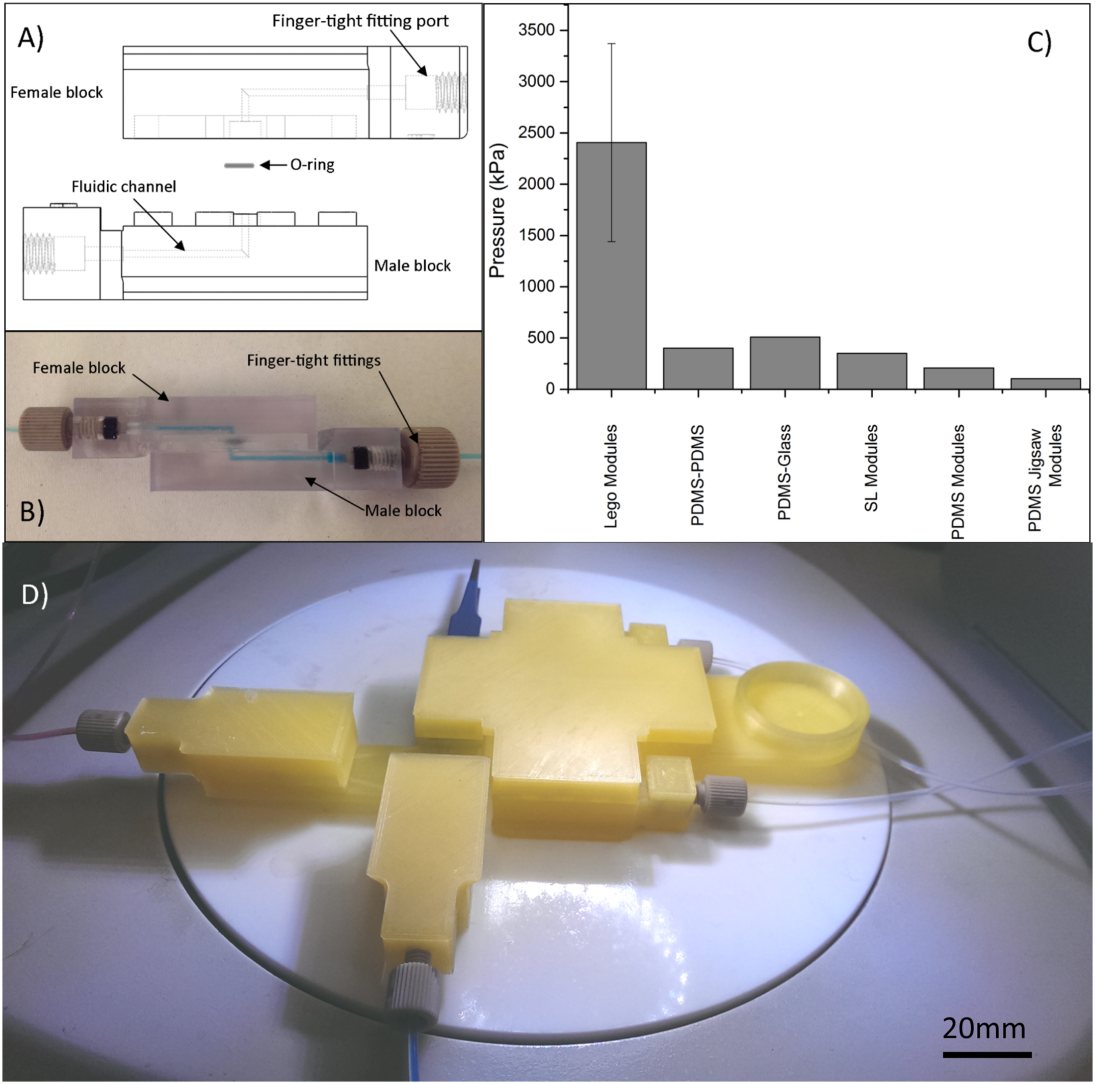
Lego^®^ like 3D printed modules. A) Profile illustration of the female (top) and male (bottom) inlet/outlet modules, when not connected together, showing the internal structure of the modules with a channel running through both modules from a threaded finger-tight fitting port. B) Photograph of two modules when connected together. Modules are connected by simply pushing them together interlocking the rounded studs on the male block into the recess in the female blocks. No form of adhesion is necessary. The modules in this image were created on a stereolithography printer for increased transparency. C) Pressure limit of the Lego^®^-like modular system, as determined experimentally. Error bars represent 95% confidence level. The pressure limit is compared to the previously reported pressures tolerances of alternative techniques: Maximum pressure of plasma bonded PDMS-PDMS and PDMS-Glass devices [43]; Stereolithography printed modules connected to a microfluidic ‘breadboard’[33]; PDMS modules secured to glass using uncured PDMS with blocks connected together using UV curable adhesive [44]; Self-aligning PDMS jigsaw modules secured to glass using uncured PDMS [45]. D) A modular system created using 7 different modules connected together without the need of any adhesive. This system features 4 inlet/outlet modules, a T-junction, a flow-focusing junction and a collection vessel module.

The accessibility and practicality of 3D printed devices can be further enhanced by using a modular system that allows users to print out modular components and connect them together to create reconfigurable microfluidic systems. To this end a modular system based on the interconnectivity of Lego^®^ blocks was developed. Using rounded studs protruding from the top surface of blocks and a reciprocal cavity on the underside enables the interlocking of modules and fluid connectivity. Fluid-tight seals can be made rapidly and repeatably between different modules (Fig 8A and 8B). An O-ring within the interconnecting port of the female module ensures a leak-free seal. To demonstrate the capabilities of the modular system, a number of modules were created that demonstrate many of the typical components of microfluidic systems such as T-junctions, mixing channels and flow-focusing junctions. Schematics of these components can be found in S2 Table. Leak-free connectivity was possible with channel diameters down to 600μm (height and width). A seven component modular system comprised of observation chamber, 4 independent fluid inputs, and sequential T-junction and flow focussing geometries for the production of fluid droplets with alternating composition can be seen in Fig 8D.

Pressure tests were performed to investigate the integrity of seal between printed PLA modules, and also the 3D printed ports (Fig 8C). The pressures the devices were able to withstand (~2000kPa+) far exceed the pressures that are required for most microfluidic applications. The pressure tolerance of the connected modules compares favourably to reported pressure tolerances for devices fabricated by traditional polydimethylsiloxane (PDMS) fabrication [43], as well as previously reported modular systems manufactured by more traditional fabrication methods [44, 45] (Fig 8C). It should be noted that in two of the pressure tests the failure was at the connection to the pump rather than between the modules. When the pressure exceeded ~2900kPa the tubing connecting the modules to the pump came loose from the finger-tight fitting in the pump whilst no leakage was observed within the modules or at the connection between them.

## Conclusions

Simple, easily manufactured 3D printed microfluidic devices have been demonstrated. Despite previous concerns, it is possible to achieve high fidelity of fluidic geometries to accurately reflect the intended design, generate transparent devices for observation and employ high pressure seals in microfluidic devices printed using cheap ‘off-the-shelf’ FFF printers. These devices are suitable for application by many researchers in the life-sciences who could benefit from microfluidic technology. This utility is demonstrated here by the encapsulation of live stem cells within alginate microspheres in such printed devices. We have also shown that multicomponent fluidic systems can be created easily by simply connecting modular interlocking blocks together. This Lego^®^-like system also ensures that the modules self-align meaning leak-free, reconfigurable microfluidic systems can be rapidly assembled and used in the lab. The use of FFF printing offers great accessibility to micro- and millifluidic fabrication as the falling costs and rising resolution of these printers makes them affordable to most laboratories. As a democratising technology, 3D print designs are easily shared between researchers by email or online repositories. This has the potential to widen accessibility further by eliminating the design barrier in addition to the fabrication barrier largely limiting access to microfluidic technology at present. To the best of the author’s knowledge, fully transparent microfluidic devices have not previously been realised by FFF printing, nor have functional flow-focussing geometries been demonstrated for droplet microfluidic applications. Additionally, the simplicity of fabrication of even complex fluidic pathways makes 3D printing highly appealing, allowing people with previously limited experience in the field to access the many advantages of microfluidic techniques. The connections between modules have been demonstrated to be capable of withstanding pressure of over 2000kPa; a pressure higher than many alternative fabrication methods and greater than required of many microfluidic applications.

It is envisaged that future development of 3D printed microfluidic devices can begin to exploit another key advantage of 3D printing; the ability to design truly three-dimensional structures. Traditional fabrication techniques have generally between limited to 2.5D planar fluid pathways but 3D printing offers the opportunity to exploit the z-dimension like never before.

## Supporting Information

S1 Fig Ethics documentation. Letter confirming permission to carry out experimentation with dental pulp stem cells from University Health Board.
(PDF)

S2 Fig Ethics documentation. South East Wales Research Ethics Committee ethical review.
(PDF)

S3 Fig Ethics documentation. NHS Patient Safety Agency ethical guidelines for research in human subjects.
(PDF)

S4 Fig 3D printer fidelity measurements. a-h) Comparisons of measured and desired dimensions of 3d printed circular channels. Dotted line indicates perfect fidelity between the CAD model and the printed channel. Equations and R^2^ value are for a linear fit of the measured data. a) Measured width of a horizontally printed channel using FFF printing with 50μm layers. b) Measured depth of a horizontally printed channel using FFF printing with 50μm layers. c) Measured width of a horizontally printed channel using FFF printing with 100μm layers. d) Measured depth of a horizontally printed channel using FFF printing with 100μm layers. e) Measured width of a horizontally printed channel using FFF printing with 25μm layers. f) Measured depth of a horizontally printed channel using FFF printing with 25μm layers. g) Measured diameter of a vertically printed channel using FFF printing. h) Measured diameter of a vertically printed channel using SL printing. i-k) Images of a 1.4mm diameter channels fabricated using 3D printing: i) Horizontally printed using FFF with 50μm layers. j) Horizontally printed using FFF with 100μm layers. k) Vertically printed using SL.
(DOCX)

S5 Fig Interferometer scan of 3D printed surface. The 1200mm/min 50μm layers test piece used in the transparency tests was sputter coated with gold and then scanned using a Veeco Interferometer. The average roughness was found to be 2.28μm whilst the peak-to-peak roughness was 1.81μm. The nature of extrusion based 3D printing creates a repeated pattern of peaks and troughs, with the peaks created at the interface between two ‘strips’ of extruded material.
(DOCX)

S6 Fig Wetting problems observed with t-glase filament. Both A and B show the same T-junction creating segments of water in oil, however B is taken approximately 5 minutes after A showing that segment breakup has moved down the channel.
(DOCX)

S7 Fig Optical transmission of 3D printed devices at various print speeds and layer heights. Maximal transmission is represented by transparent PLA that is melted in an oven such that it is the same thickness as the printed devices.
(DOCX)

S1 Table Print settings used to produce transparent microfluidic flow focusing device for encapsulating stem cells. Slicer parameters are left as determined by high quality auto-configuration in Simplify3D 2.2.2 unless stated below.
(DOCX)

S2 Table Library of Lego^®^ based microfluidic modules that have been designed and printed. Each module is named (left), shown in 3D CAD form (centre) and finally the internal structures are shown (right). Internal boundaries are depicted with a dotted blue line.
(DOCX)

S1 Video Stem cells imaged under flow conditions in a transparent 3D printed PLA device as depicted in Fig 5D–5F. (AVI)

S2 Video Aqueous slug containing 250 nM sulforhodamine b imaged flowing through the channel of a transparent 3D printed PLA device as depicted in Fig 5D–5F. (AVI)

S3 Video Aqueous solution containing 25 nM sulforhodamine b imaged entering the channel of a transparent 3D printed PLA device as depicted in Fig 5D–5F. (AVI)
