# Supplementary material for: Simple and Versatile 3D Printed Microfluidics Using Fused Filament Fabrication
Source: PLoS One. 2016 Apr 6;11(4):e0152023. doi: 10.1371/journal.pone.0152023 (PMC4822857; doi:10.1371/journal.pone.0152023)
Supplement: S3 Fig — NHS Patient Safety Agency ethical guidelines for research in human subjects. (PDF) [file pone.0152023.s003.pdf]

2

National Patient Safety Agency

National Research Ethics Service

RESEARCH IN HUMAN SUBJECTS OTHER THAN CLINICAL TRIALS OF INVESTIGATIONAL MEDICINAL PRODUCTS

After ethical review – guidance for sponsors and investigators

This document sets out important guidance for sponsors and investigators on the conduct and management of research with a favourable opinion from a NHS Research Ethics Committee. Please read the guidance carefully. A failure to follow the guidance could lead to the committee reviewing its opinion on the research.

1. Further communications with the Research Ethics Committee

1.1 Further communications during the research with the Research Ethics Committee that gave the favourable ethical opinion (hereafter referred to in this document as "the Committee") are the personal responsibility of the Chief Investigator.

2. Commencement of the research

2.1 It is assumed that the research will commence within 12 months of the date of the favourable ethical opinion.

2.2 The research must not commence at any site until the local Principal Investigator (PI) or research collaborator has obtained management permission or approval from the organisation with responsibility for the research participants at the site.

2.3 Should the research not commence within 12 months, the Chief Investigator should give a written explanation for the delay

2.4 Should the research not commence within 24 months, the Committee may review its opinion.

3. Duration of ethical approval

3.1 The favourable opinion for the research generally applies for the duration of the research. If it is proposed to extend the duration of the study as specified in the application form, the Committee should be notified.

3.2 Where the research involves the use of "relevant material" for the purposes of the Human Tissue Act 2004, authority to hold the material under the terms of the ethical approval applies until the end of the period declared in the application and approved by the Committee.

4. <u>Progress reports</u>

4.1 Research Ethics Committees are expected to keep a favourable opinion under review in the light of progress reports and any developments in the study. The Chief Investigator should submit a progress report to the Committee 12 months after the date on which the favourable opinion was given. Annual progress reports should be submitted thereafter.

4.2 Progress reports should be in the format prescribed by NRES and published on the website (see www.nres.npsa.nhs.uk/applicants/after-ethical-review/).

4.3 The Chief Investigator may be requested to attend a meeting of the Committee or Sub-Committee to discuss the progress of the research.

5. <u>Amendments</u>

5.1 If it is proposed to make a substantial amendment to the research, the Chief Investigator should submit a notice of amendment to the Committee.

5.2 A substantial amendment is any amendment to the terms of the application for ethical review, or to the protocol or other supporting documentation approved by the Committee, that is likely to affect to a significant degree:

(a) the safety or physical or mental integrity of the trial participants
(b) the scientific value of the trial
(c) the conduct or management of the trial.

5.3 Notices of amendment should be in the format prescribed by NRES and published on the website, and should be personally signed by the Chief Investigator. The agreement of the sponsor should be sought before submitting the notice of amendment.

5.4 A substantial amendment should not be implemented until a favourable ethical opinion has been given by the Committee, unless the changes to the research are urgent safety measures (see section 7). The Committee is required to give an opinion within 35 days of the date of receiving a valid notice of amendment.

5.5 Amendments that are not substantial amendments ("minor amendments") may be made at any time and do not need to be notified to the Committee.

6. <u>Changes to sites</u>

Management permission (all studies)

6.1 For all studies, management permission should be obtained from the host organisation where it is proposed to:

- include a new site in the research, not included in the list of proposed research sites in the original REC application
- appoint a new PI or Local Collaborator at a research site
- make any other significant change to the conduct or management of a research site.

In the case of any new NHS site, the Site-Specific Information (SSI) Form should be submitted to the R&D office for review as part of the R&D application.

Site-specific assessment (where required)

6.2 The following guidance applies only to studies requiring site-specific assessment (SSA) as part of ethical review.

6.3 In the case of *NHS/HSC sites*, SSA responsibilities are undertaken on behalf of the REC by the relevant R&D office as part of the research governance review. The Committee's favourable opinion for the study will apply to any new sites and other changes at sites provided that management permission is obtained. There is no need to notify the Committee (or any other REC) about new sites or other changes, or to provide a copy of the SSI Form.

6.4 Changes at *non-NHS sites* require review by the local REC responsible for site-specific assessment (SSA REC). Please submit the SSI Form (or revised SSI Form as appropriate) to the SSA REC together with relevant supporting documentation. The SSA REC will advise the main REC whether it has any objection to the new site/PI or other change. The main REC will notify the Chief Investigator and sponsor of its opinion within a maximum of 35 days from the date on which a valid SSA application has been received by the SSA REC.

Studies not requiring SSA

6.5 For studies designated by the Committee as not requiring SSA, there is no requirement to notify the Committee of the inclusion of new sites or other changes at sites, either for NHS or non-NHS sites. However, management permission should still be obtained from the responsible host organisation (see 6.1 above).

7. Urgent safety measures

7.1 The sponsor or the Chief Investigator, or the local Principal Investigator at a trial site, may take appropriate urgent safety measures in order to protect research participants against any immediate hazard to their health or safety.

7.2 The Committee must be notified within three days that such measures have been taken, the reasons why and the plan for further action.

8. Serious Adverse Events

8.1 A Serious Adverse Event (SAE) is an untoward occurrence that:

(a) results in death
(b) is life-threatening
(c) requires hospitalisation or prolongation of existing hospitalisation
(d) results in persistent or significant disability or incapacity
(e) consists of a congenital anomaly or birth defect
(f) is otherwise considered medically significant by the investigator.

8.2 A SAE occurring to a research participant should be reported to the Committee where in the opinion of the Chief Investigator the event was related to administration of any of the research procedures, and was an unexpected occurrence.

8.3 Reports of SAEs should be provided to the Committee within 15 days of the Chief Investigator becoming aware of the event, in the format prescribed by NRES and published on the website.

8.4 The Chief Investigator may be requested to attend a meeting of the Committee or Sub-Committee to discuss any concerns about the health or safety of research subjects.

8.5 Reports should not be sent to other RECs in the case of multi-site studies.

9. <u>Conclusion or early termination of the research</u>

9.1 The Chief Investigator should notify the Committee in writing that the research has ended within 90 days of its conclusion. The conclusion of the research is defined as the final date or event specified in the protocol, not the completion of data analysis or publication of the results.

9.2 If the research is terminated early, the Chief Investigator should notify the Committee within 15 days of the date of termination. An explanation of the reasons for early termination should be given.

9.3 Reports of conclusion or early termination should be submitted in the form prescribed by NRES and published on the website.

10. <u>Final report</u>

10.1 A summary of the final report on the research should be provided to the Committee within 12 months of the conclusion of the study. This should include information on whether the study achieved its objectives, the main findings, and arrangements for publication or dissemination of the research including any feedback to participants.

11. <u>Review of ethical opinion</u>

11.1 The Committee may review its opinion at any time in the light of any relevant information it receives.

11.2 The Chief Investigator may at any time request that the Committee reviews its opinion, or seek advice from the Committee on any ethical issue relating to the research.
