# Supplementary material for: Simple and Versatile 3D Printed Microfluidics Using Fused Filament Fabrication
Source: PLoS One. 2016 Apr 6;11(4):e0152023. doi: 10.1371/journal.pone.0152023 (PMC4822857; doi:10.1371/journal.pone.0152023)
Supplement: S4 Fig — a-h) Comparisons of measured and desired dimensions of 3d printed circular channels. Dotted line indicates perfect fidelity between the CAD model and the printed channel. Equations and R2 value are for a linear fit of the measured data. a) Measured width of a horizontally printed channel using FFF printing with 50μm layers. b) Measured depth of a horizontally printed channel using FFF printing with 50μm layers. c) Measured width of a horizontally printed channel using FFF printing with 100μm layers. d) Measured depth of a horizontally printed channel using FFF printing with 100μm layers. e) Measured width of a horizontally printed channel using FFF printing with 25μm layers. f) Measured depth of a horizontally printed channel using FFF printing with 25μm layers. g) Measured diameter of a vertically printed channel using FFF printing. h) Measured diameter of a vertically printed channel using SL printing. i-k) Images of a 1.4mm diameter channels fabricated using 3D printing: i) Horizontally printed using FFF with 50μm layers. j) Horizontally printed using FFF with 100μm layers. k) Vertically printed using SL. (DOCX) [file pone.0152023.s004.docx]

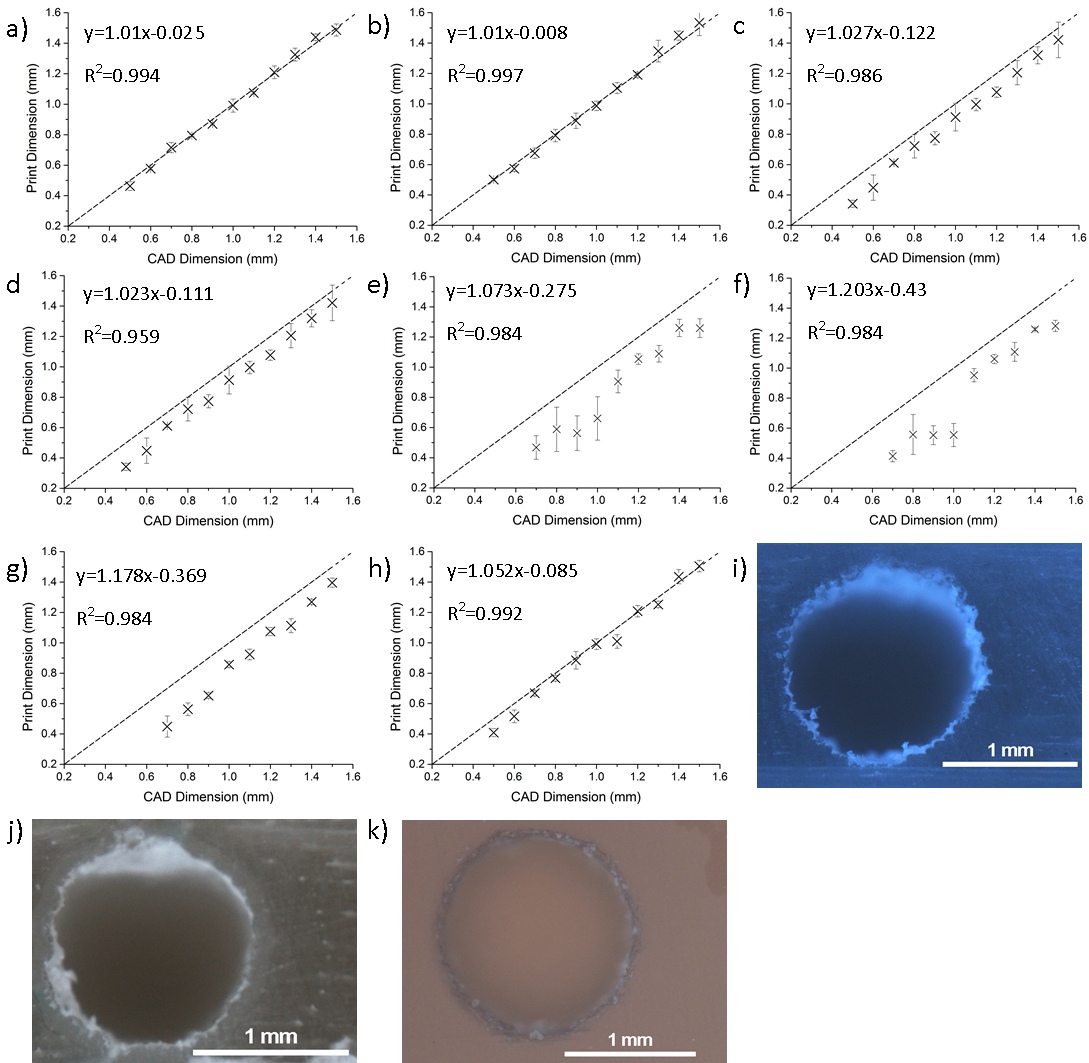


**S4 Fig. 3D printer fidelity measurements.** a-h) Comparisons of measured and desired dimensions of 3d printed circular channels. Dotted line indicates perfect fidelity between the CAD model and the printed channel. Equations and R^2^ value are for a linear fit of the measured data. a) Measured width of a horizontally printed channel using FFF printing with 50µm layers. b) Measured depth of a horizontally printed channel using FFF printing with 50µm layers. c) Measured width of a horizontally printed channel using FFF printing with 100µm layers. d) Measured depth of a horizontally printed channel using FFF printing with 100µm layers. e) Measured width of a horizontally printed channel using FFF printing with 25µm layers. f) Measured depth of a horizontally printed channel using FFF printing with 25µm layers. g) Measured diameter of a vertically printed channel using FFF printing. h) Measured diameter of a vertically printed channel using SL printing. i-k) Images of a 1.4mm diameter channels fabricated using 3D printing: i) Horizontally printed using FFF with 50µm layers. j) Horizontally printed using FFF with 100µm layers. k) Vertically printed using SL.

Reasonably good print fidelity can be achieved using 50µm layers, as can be seen in S4A and S4B Figs. Print fidelity is less accurate when the channels are printed perpendicular to the print bed (S4G Fig). This should be taken into account when designing devices. S4H Fig demonstrates that the fidelity of FFF printed devices can be favourably compared to that of SL printing; both techniques can achieve relatively high print accuracy. S4C and S4D Figs highlight that 100µm layers give poorer fidelity but the inaccuracy appears to be consistent so it is possible to design around these inaccuracies. 100µm layer printing is twice as fast as 50µm printing so there is a balance to be struck between speed and accuracy that a user must consider when printing. The accuracy was found to be poor when 25µm layers were used (S4 Figs E and F).
