# Supplementary material for: Simple and Versatile 3D Printed Microfluidics Using Fused Filament Fabrication
Source: PLoS One. 2016 Apr 6;11(4):e0152023. doi: 10.1371/journal.pone.0152023 (PMC4822857; doi:10.1371/journal.pone.0152023)
Supplement: S5 Fig — The 1200mm/min 50μm layers test piece used in the transparency tests was sputter coated with gold and then scanned using a Veeco Interferometer. The average roughness was found to be 2.28μm whilst the peak-to-peak roughness was 1.81μm. The nature of extrusion based 3D printing creates a repeated pattern of peaks and troughs, with the peaks created at the interface between two ‘strips’ of extruded material. (DOCX) [file pone.0152023.s005.docx]

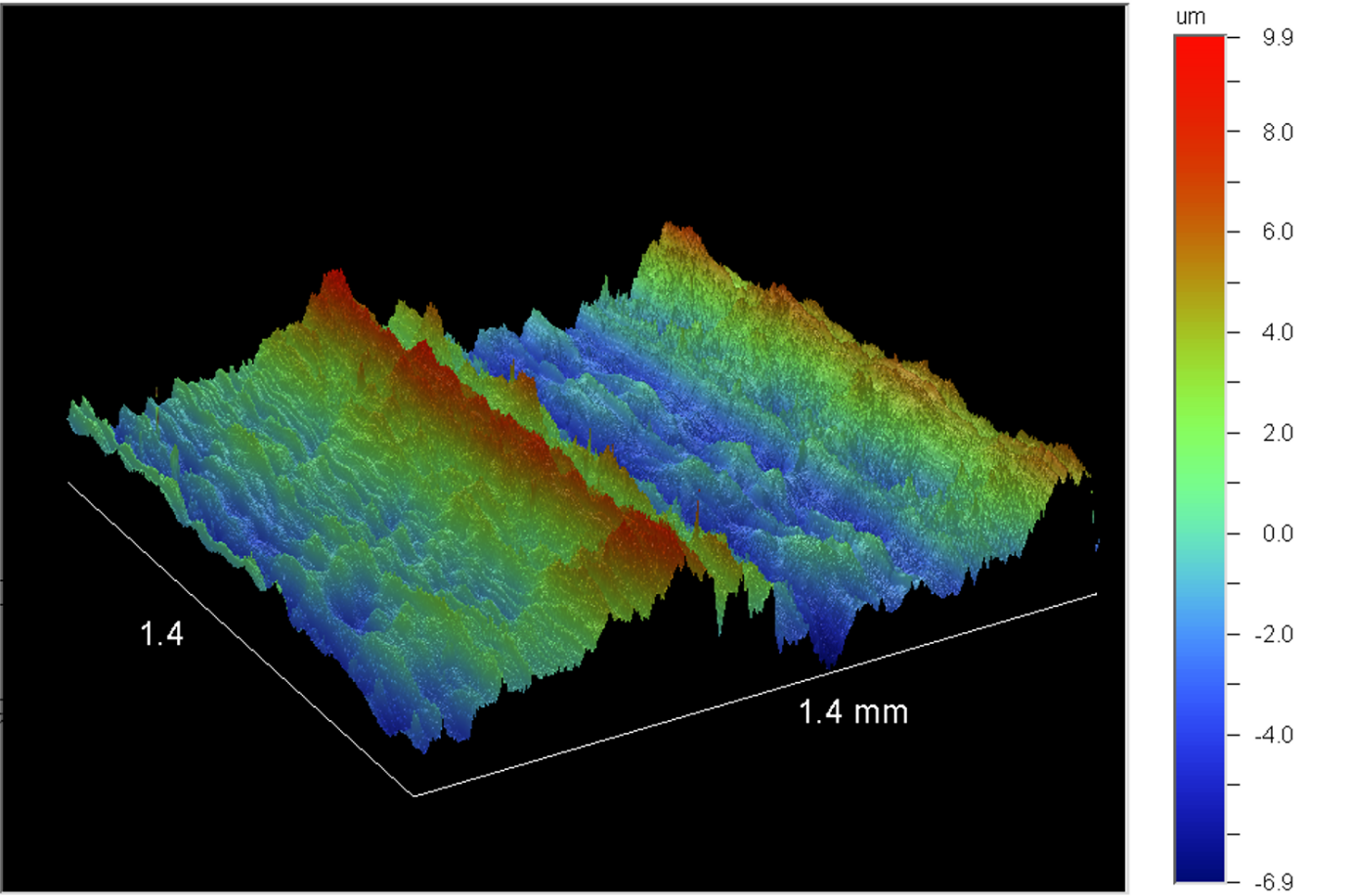


**S5 Fig. Interferometer scan of 3D printed surface.** The 1200mm/min 50µm test piece used in the transparency tests was sputter coated with gold and then scanned using a Veeco Interferometer. The average roughness was found to be 2.28µm whilst the peak-to-peak roughness was 1.81µm. The nature of extrusion based 3D printing creates a repeated pattern of peaks and troughs, with the peaks created at the interface between two ‘strips’ of extruded material.
