# Supplementary material for: Simple and Versatile 3D Printed Microfluidics Using Fused Filament Fabrication
Source: PLoS One. 2016 Apr 6;11(4):e0152023. doi: 10.1371/journal.pone.0152023 (PMC4822857; doi:10.1371/journal.pone.0152023)
Supplement: S6 Fig — Both A and B show the same T-junction creating segments of water in oil, however B is taken approximately 5 minutes after A showing that segment breakup has moved down the channel. (DOCX) [file pone.0152023.s006.docx]

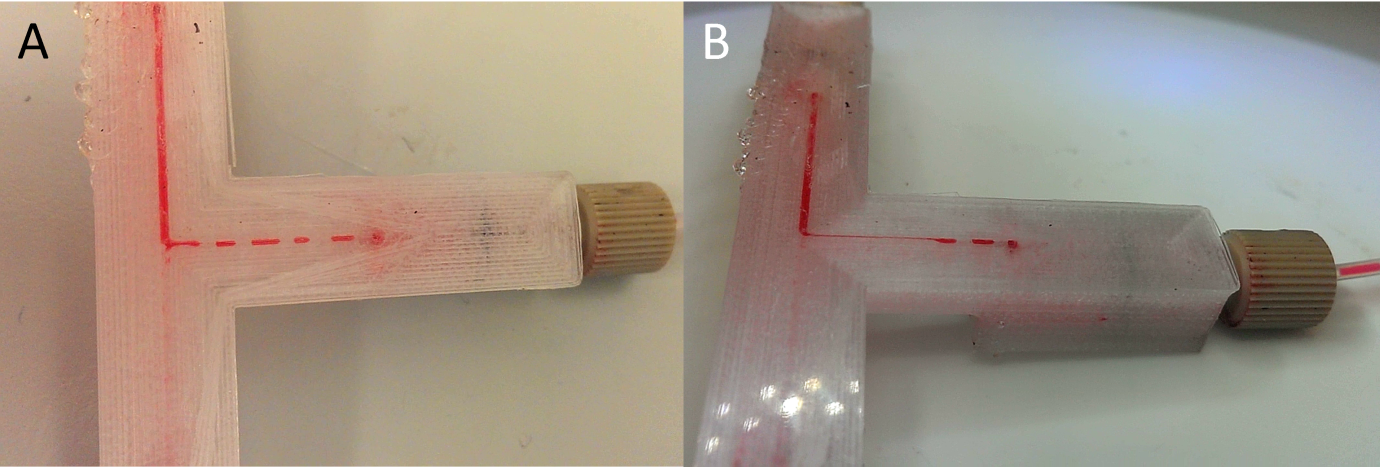


**S6 Fig. Wetting problems observed with t-glase filament.** Both A and B show the same T-junction creating segments of water in oil, however B is taken approximately 5 minutes after A showing that segment breakup has moved down the channel.

S6 Fig shows the changing wetting characteristics that were observed with ABS and PET filaments. Fluid absorption causes the segment breakup position to move down the channel over time. This was not a problem with PLA material used to optimise transparency of functional 3D printed devices.
