# Supplementary material for: Simple and Versatile 3D Printed Microfluidics Using Fused Filament Fabrication
Source: PLoS One. 2016 Apr 6;11(4):e0152023. doi: 10.1371/journal.pone.0152023 (PMC4822857; doi:10.1371/journal.pone.0152023)
Supplement: S7 Fig — Maximal transmission is represented by transparent PLA that is melted in an oven such that it is the same thickness as the printed devices. (DOCX) [file pone.0152023.s007.docx]

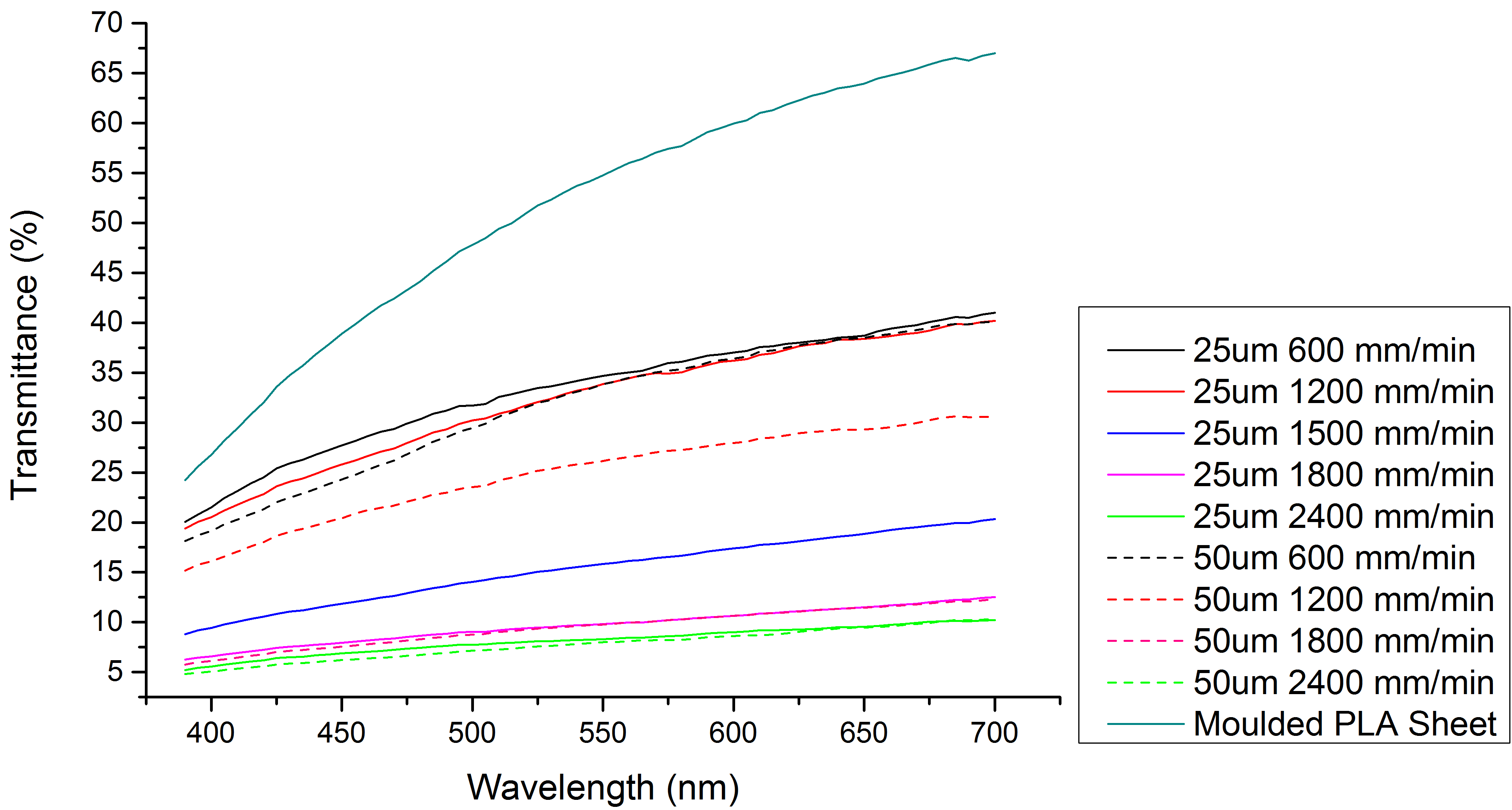


**S7 Fig. Optical transmission of 3D printed devices at various print speeds and layer heights.** Maximal transmission is represented by transparent PLA that is melted in an oven such that it is the same thickness as the printed devices
