# Supplementary material for: Simple and Versatile 3D Printed Microfluidics Using Fused Filament Fabrication
Source: PLoS One. 2016 Apr 6;11(4):e0152023. doi: 10.1371/journal.pone.0152023 (PMC4822857; doi:10.1371/journal.pone.0152023)
Supplement: S1 Table — Slicer parameters are left as determined by high quality auto-configuration in Simplify3D 2.2.2 unless stated below. (DOCX) [file pone.0152023.s008.docx]

**S1 Table. Print settings used to produce transparent microfluidic flow focusing device for encapsulating stem cells**. Slicer parameters are left as determined by high quality auto-configuration in Simplify3D 2.2.2 unless stated below.

| **Printer** | Ultimaker 2 |
| --- | --- |
| **Slicer** | Simplify3D 2.2.2 |
| **Material** | Faberdashery Crystal Clear PLA 3mm |
| **Layer Height** | 50µm |
| **Fill Pattern** | Rectilinear |
| **Infill Percentage** | 100% |
| **Infill Angles** | 0°, 90° |
| **Infill Extrusion Width** | 125% |
| **Printing Speed** | 1800 mm/min |
| **Extruder Temperature** | 215°C |
