# Supplementary material for: Simple and Versatile 3D Printed Microfluidics Using Fused Filament Fabrication
Source: PLoS One. 2016 Apr 6;11(4):e0152023. doi: 10.1371/journal.pone.0152023 (PMC4822857; doi:10.1371/journal.pone.0152023)
Supplement: S2 Table — Each module is named (left), shown in 3D CAD form (centre) and finally the internal structures are shown (right). Internal boundaries are depicted with a dotted blue line. (DOCX) [file pone.0152023.s009.docx]

**S2 Table. Library of Lego® based microfluidic modules that have been designed and printed.** Each module is named (left), shown in 3D CAD form (centre) and finally the internal structures are shown (right). Internal boundaries are depicted with a dotted blue line. Printable STL files are available from the authors upon request.

| Male Inlet/Outlet | 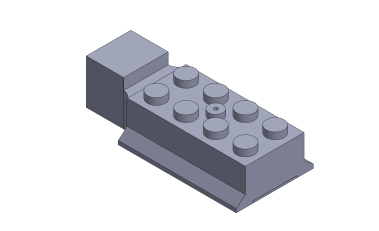 | 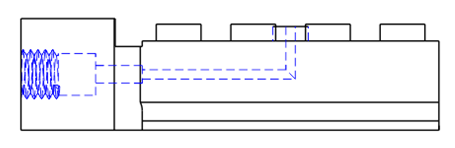 |
| --- | --- | --- |
| Female Inlet/Outlet | 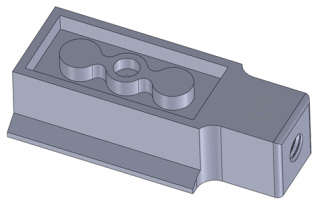 | 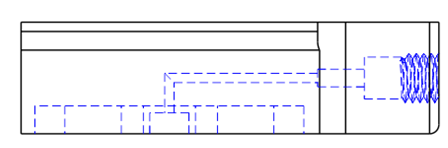 |
| Flow-Focusing Junction w/Observation Window | 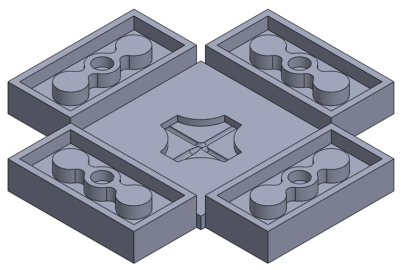 | 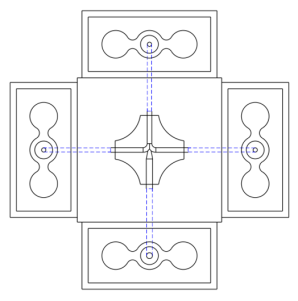 |
| Mixer/Curer | 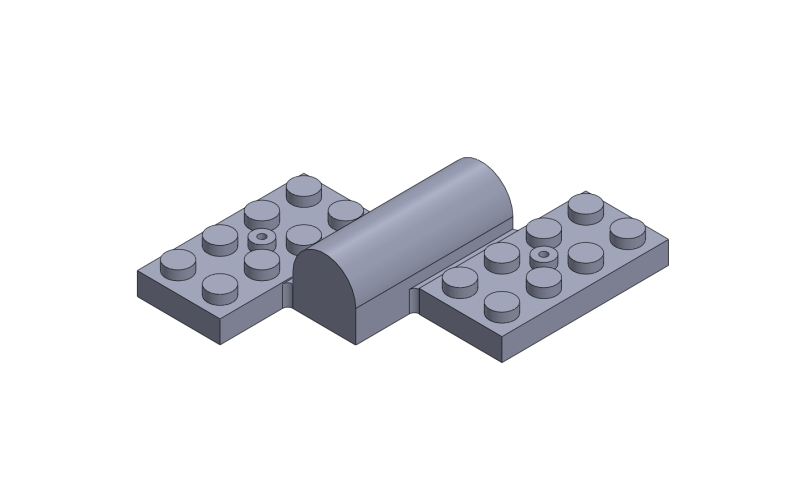 | 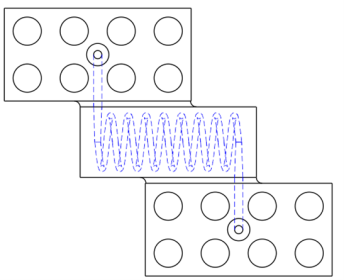 |
| UV Curer | 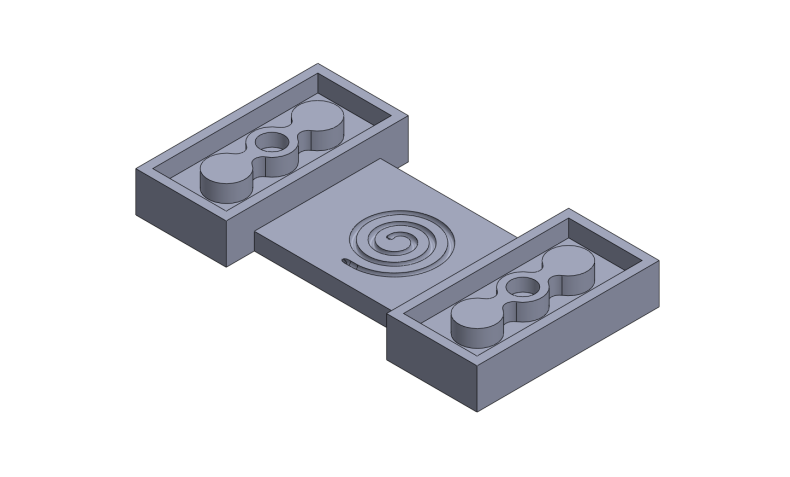 | 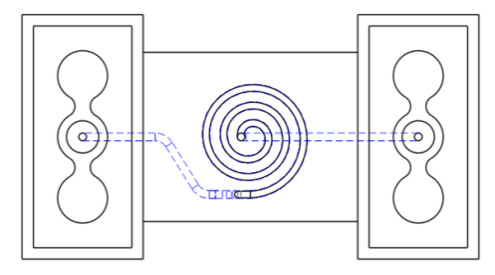 |
| Observation Module | 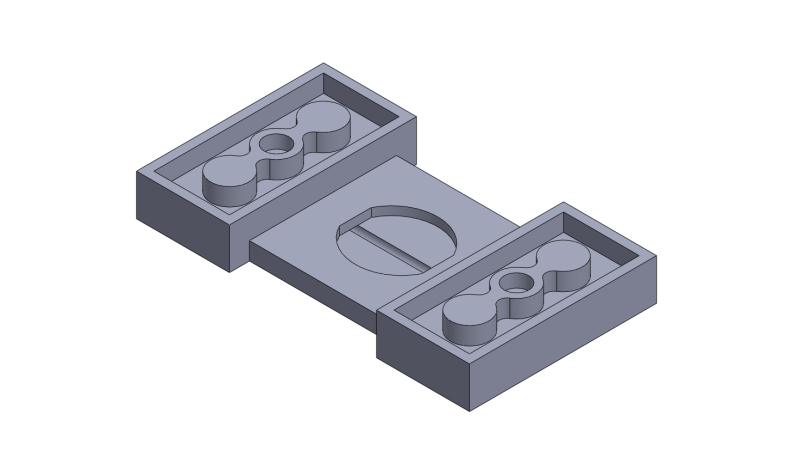 | 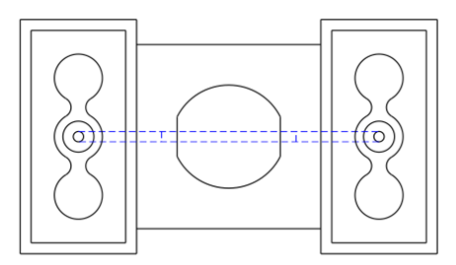 |
| Flow-Focusing Junction | 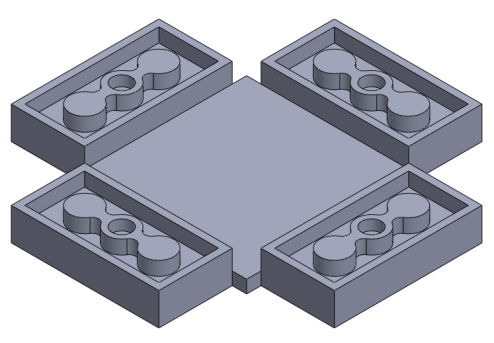 | 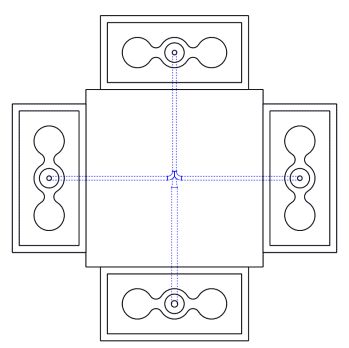 |
| Collection Vessel | 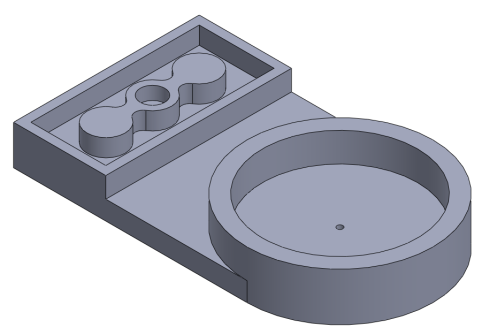 | 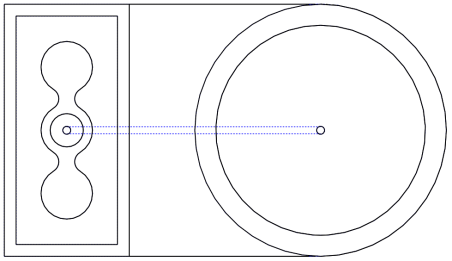 |
| T-Junction | 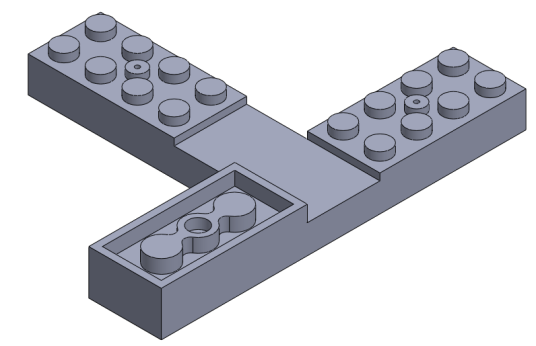 | 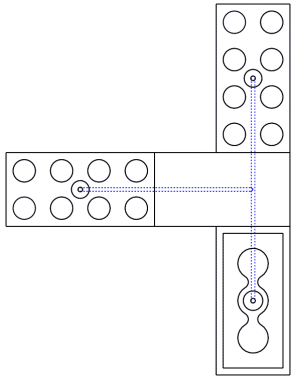 |
| 2x2 Male Inlet | 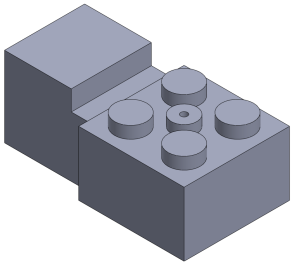 | 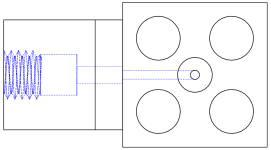 |
| 2x2 Female Outlet | 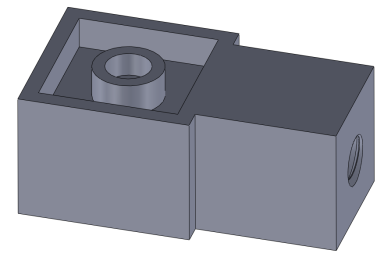 | 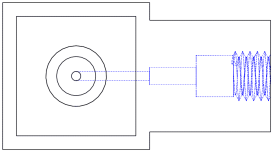 |
| 2x2 Connector  T- Junction | 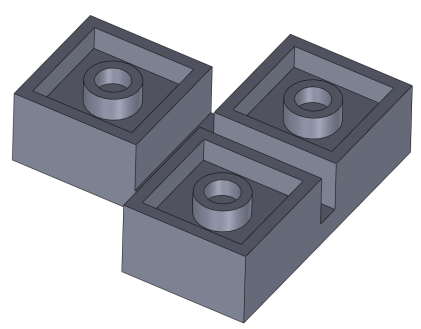 | 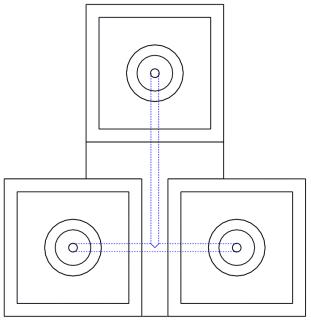 |
