# Supplementary material for: Simple and Versatile 3D Printed Microfluidics Using Fused Filament Fabrication
Source: PLoS One. 2016 Apr 6;11(4):e0152023. doi: 10.1371/journal.pone.0152023 (PMC4822857; doi:10.1371/journal.pone.0152023)
Supplement: S1 Fig — Letter confirming permission to carry out experimentation with dental pulp stem cells from University Health Board. (PDF) [file pone.0152023.s001.pdf]

GIG CYMRU NHS WALES | Bwrdd Iechyd Prifysgol Caerdydd a'r Fro
Cardiff and Vale University Health Board

**Ysbyty Athrofaol Cymru**
**University Hospital of Wales**

Heath Park,
Cardiff, CF14 4XW
Phone 029 2074 7747
Fax 029 2074 3838
Minicom 029 2074 3632

Parc Y Mynydd Bychan,
Caerdydd, CF14 4XW
Ffôn 029 2074 7747
Ffacs 029 2074 3838
Minicom 029 2074 3632

Eich cyf/Your ref
Ein cyf/Our ref
Welsh Health Telephone Network 1872
Direct line/Llinell uniongyrchol

Tel: 029 20746986
Fax: 029 20745311
CAV_Research.Development@wales.nhs.uk

From: Professor JI Bisson
R&D Director
R&D Office, 2nd Floor TB2
University Hospital of Wales
Cardiff
CF14 4XW

03 May 2012

Dr Alastair Sloan
Cardiff University
School of Dentistry
Heath Park
Cardiff
CF14 4XY

Dear Dr Sloan

**Cardiff and Vale UHB Ref : 12/DEN/5348 : Harnessing Dental Pulp Cells To Enhance Dental Tissue Regeneration**

**NISCHR PCU Ref: 80343**

The above project was forwarded to Cardiff and Vale University Health Board R&D Office by the NISCHR Permissions Coordinating Unit. A Governance Review has now been completed on the project.

Documents approved for use in this study are:

| **Document** | **Version** | **Date** |
|---|---|---|
| NHS R&D Form | 3.4 | Received 5 April 2012 |
| NHS SSI Form | 3.4 | Received 5 April 2012 |
| Protocol | 0.6 | 27 February 2012 |
| Study Flow Chart | 0.4 | 14 February 2012 |
| Participant Information Sheet | 0.4 | 16 April 2012 |
| Participant Information Sheet: Questionnaire | 0.47 | 16 April 2012 |
| Reminder Letter | 0.3 | 16 April 2012 |
| Interview Confirmation Letter | 0.2 | 16 April 2012 |
| Introductory Letter – Questionnaire | 0.2 | 16 April 2012 |
| Questionnaire: Pre-procedure | 0.7 | 16 April 2012 |
| Questionnaire: Post-procedure | 0.5 | 16 April 2012 |



Bwrdd Iechyd Prifysgol Caerdydd a'r Fro yw enw gweithredol Bwrdd Iechyd Lleol Prifysgol Caerdydd a'r Fro
Cardiff and Vale University Health Board is the operational name of Cardiff and Vale University Local Health Board

POSITIVE ABOUT DISABLED PEOPLE

UHW129X

| | | |
|---|---|---|
| Interview Topic Guide | 1.0 | 03 April 2012 |
| Consent Form: Questionnaire | 0.5 | 16 April 2012 |
| Consent Form: Interview | 0.3 | 16 April 2012 |

I am pleased to inform you that the UHB has no objection to your proposal.

You have informed us that Cardiff University is willing to act as Sponsor under the Research Governance Framework for Health and Social Care.

Your study may be eligible for adoption by the NISCHR Portfolio. You must now apply to have this trial adopted in order for the UHB to receive funding to support this study. If your study is adopted onto the NISCHR Clinical Research Portfolio (CRP), it will be a condition of this NHS research permission, that you will be required to regularly upload recruitment data onto the portfolio database.

To apply for adoption onto the NISCHR CRP, please go to: http://www.wales.nhs.uk/sites3/page.cfm?orgid=580&pid=31979 Once adopted, NISCHR CRP studies may be eligible for additional support through the NISCHR Clinical Research Centre. Further information can be found at: http://www.wales.nhs.uk/sites3/page.cfm?orgid=580&pid=28571

To upload recruitment data, please follow this link: http://www.crncc.nihr.ac.uk/about_us/processes/portfolio/p_recruitment Uploading recruitment data will enable NISCHR to monitor research activity within NHS organizations, leading to NHS R&D allocations which are activity driven. Uploading of recruitment data will be monitored by your colleagues in the R&D office. If you need any support in uploading this data, please contact CAV_research.development@wales.nhs.uk

Please accept this letter as confirmation of permission for the project to begin within this UHB.

May I take this opportunity to wish you success with the project and remind you that as Principal Investigator you are required to:

- Inform NISCHR PCU and the UHB R&D Office if any external or additional funding is awarded for this project in the future
- Submit any substantial amendments relating to the study to NISCHR PCU in order that they can be reviewed and approved prior to implementation
- Ensure NISCHR PCU is notified of the study's closure
- Ensure that the study is conducted in accordance with all relevant policies, procedures and legislation
- Provide information on the project to the UHB R&D Office as requested from time to time, to include participant recruitment figures

Yours sincerely,

**Professor Jonathan I Bisson**
**R&D Director**

**CC R&D Lead Prof Vaseekaran Sivarajasingam**
**CC Sponsor contact, Chris Shaw, RACD**
**CC Data Manager / Study Coordinator, Kathleen Withers, CEDAR**
