# Supplementary material for: Simple and Versatile 3D Printed Microfluidics Using Fused Filament Fabrication
Source: PLoS One. 2016 Apr 6;11(4):e0152023. doi: 10.1371/journal.pone.0152023 (PMC4822857; doi:10.1371/journal.pone.0152023)
Supplement: S2 Fig — South East Wales Research Ethics Committee ethical review. (PDF) [file pone.0152023.s002.pdf]

Part of the research infrastructure for Wales funded by the National Institute for Social Care and Health Research, Welsh Government.
Yn rhan o seilwaith ymchwil Cymru a ariannir gan y Sefydliad Cenedlaethol ar gyfer Ymchwil Gofal Cymdeithasol ac Iechyd, Llywodraeth Cymru

NISCHR

Gwasanaeth Moeseg Ymchwil | RES | Research Ethics Service

South East Wales Research Ethics Committee, Panel B
Sixth Floor, Churchill House
17 Churchill Way
Cardiff CF10 2TW
Telephone : 029 2037 6823

E-mail : carl.phillips@wales.nhs.uk
Website : www.nres.nhs.uk

20 April 2012

Dr Alastair Sloan
Reader in Bone Biology & Tissue Engineering
Cardiff University
School of Dentistry
Cardiff University
Heath Park
CF14 4XY

Dear Dr Sloan

**Study title:**	**Harnessing Dental Pulp Stem Cells to Enhance Dental Tissue Regeneration**
**REC reference:**	**12/WA/0107**

The Research Ethics Committee reviewed the above application at the meeting held on the 18 April 2012.

The Committee was most grateful to Dr J Colombo for kindly taking the time to attend the meeting. The additional information and clarification that Dr Colombo was able to provide was most helpful and much appreciated.

Ethical opinion

The Committee noted that this was a single site study limited to working with newly obtained human tissue samples which would be undertaken with the aim of using the donated samples to create a material which in turn would be tested in a variety of dental materials for its ability to promote dentine formation.

The Committee in noting that the study would involve human tissue samples pointed out that when reviewing research involving human tissue, the role of the REC was to give an ethical opinion rather than to apply the law. It was the responsibility of the Chief Investigator to seek his or her own legal advice and/or to consult the Human Tissue Authority for advice where appropriate.

The Committee in noting that the study was sponsored by Cardiff University also noted that evidence of indemnity to cover any potential liability arising from the research had been provided as required by *Section 1.45* of the *Standard Operating Procedures for Research Ethics Committees version 5.1 dated March 2012,* issued by the *National Research Ethics Service (NRES).*

The Committee noted that the sponsor's representative had declared that an appropriate process of scientific critique had demonstrated that this research proposal was worthwhile and of high scientific quality. Members also noted that scientific review had been undertaken by the Medical Research Council, who had agreed to fund the study.

The Committee noted that the study would recruit as "*broadly as possible within the inclusion criteria*". Members also noted that the end of the study would be when *"sufficient teeth have been collected"* and were grateful to Dr Colombo for confirming that the study was scheduled to end in March 2013, and that it would use as many samples as were donated during that time.

The Committee noted that potential participants would initially be identified and approached regarding the study by staff of the Dental Hospital involved in their treatment and care.

The Committee noted that consent would be sought whilst patients were in the waiting area immediately prior to their tooth extraction. Whilst the REC would normally expect potential participants to be given a minimum of 24 hours in which to consider whether or not to take part in research, members agreed that in the context of this particular study the proposed consent process was appropriate.

The Committee noted that potential participants would be provided with written information about the purpose of the study, why they had been invited to participate, who was conducting the research, how the data would be used and what participation would be required of them. They would also be given the opportunity to ask any questions about the study. Written consent would be obtained prior to participation in the study and it was made clear that participation was entirely voluntary and that those taking part could withdraw at any point for any reason.

The Committee noted that there were no additional risks to participants from taking part as tooth extraction would take place regardless of participation in the study, and members further noted that participants would be invited to take part in the study whilst in the waiting room prior to their tooth extraction.

The Committee noted that the donated tissue used in the study would be human teeth which would be stored in fully anonymised form. Members noted that the study would involve the analysis or use of human DNA in the samples and that the research would not produce findings of clinical significance for donors or their relatives.

The Committee noted the assurance provided in the application form that the materials retained at the end of the study would not be *"relevant material"* as defined for the purposes of the Human Tissue Act,

The Committee noted from *section A43* of the *application form* that personal data would be stored for less than 3 months after the study had ended and pointed out that it was the responsibility of the Chief Investigator to be up to date and to comply with the requirements of the law and relevant guidelines relating to security and confidentiality of patient or other personal data, including the need to register when necessary with the appropriate Data Protection Officer.

The members of the Committee present gave a favourable ethical opinion of the above research on the basis described in the application form, protocol and supporting documentation, subject to the conditions specified below.

Ethical review of research sites

NHS Sites

The favourable opinion applies to all NHS sites taking part in the study, subject to management permission being obtained from the NHS/HSC R&D office prior to the start of the study (see "Conditions of the favourable opinion" below).

Conditions of the favourable opinion

The favourable opinion is subject to the following conditions being met prior to the start of the study.

- Management permission or approval must be obtained from each host organisation prior to the start of the study at the site concerned.
- Management permission ("R&D approval") should be sought from all NHS organisations involved in the study in accordance with NHS research governance arrangements.
- Guidance on applying for NHS permission for research is available in the Integrated Research Application System or at http://www.rdforum.nhs.uk.
- Where a NHS organisation's role in the study is limited to identifying and referring potential participants to research sites ("participant identification centre"), guidance should be sought from the R&D office on the information it requires to give permission for this activity.
- For non-NHS sites, site management permission should be obtained in accordance with the procedures of the relevant host organisation.
- Sponsors are not required to notify the Committee of approvals from host organisations
- It is responsibility of the sponsor to ensure that all the conditions are complied with before the start of the study or its initiation at a particular site (as applicable).

Approved documents

The documents reviewed and approved at the meeting were:

Document	Version	Date
Covering Letter	J S Colombo	30 March 2012
Evidence of insurance or indemnity	Zurich Municipal/Cardiff University	06 July 2011
Investigator CV	A J Sloan	15 February 2012
Letter from Sponsor	Cardiff University	19 January 2012
Other: Letter from MRC confirming grant award	MRC	19 May 2010
Participant Consent Form	1.0	16 February 2012
Participant Information Sheet	1	01 March 2012
Protocol	1.2	19 March 2012
REC application	3.1	02 April 2012

Membership of the Committee

The members of the Ethics Committee who were present at the meeting are listed on the attached sheet.

Statement of compliance

The Committee is constituted in accordance with the Governance Arrangements for Research Ethics Committees and complies fully with the Standard Operating Procedures for Research Ethics Committees in the UK.

After ethical review

Reporting requirements

The attached document "After ethical review – guidance for researchers" gives detailed guidance on reporting requirements for studies with a favourable opinion, including:

- Notifying substantial amendments
- Adding new sites and investigators
- Notification of serious breaches of the protocol
- Progress and safety reports
- Notifying the end of the study

The NRES website also provides guidance on these topics, which is updated in the light of changes in reporting requirements or procedures.

Feedback

You are invited to give your view of the service that you have received from the National Research Ethics Service and the application procedure. If you wish to make your views known please use the feedback form available on the website.

Further information is available at National Research Ethics Service website > After Review

**12/WA/0107**	**Please quote this number on all correspondence**

With the Committee's best wishes for the success of this project

Yours sincerely

Mrs S Warrell
Alternate Vice-Chair, Panel B
South East Wales Research Ethics Committees
Email: Carl.Phillips@wales.nhs.uk

Enclosures: List of names and professions of members who were present at the meeting and those who submitted written comments

"After ethical review – guidance for researchers" [SL-AR2]

Copied:- Dr John S Colombo – colombojs@cf.ac.uk

R&D for Cardiff University (resgov@cardiff.ac.uk)

Professor Barbara Chadwick, Cardiff University – ChadwickBL@cardiff.ac.uk

SloanAJ@cf.ac.uk

South East Wales Research Ethics Committee Panel B

Attendance at Committee meeting on 18 April 2012

Committee Members:

Name	Profession	Present	Notes
Professor A Bayer	Clinical Senior Lecturer & Head of Section	No	
Ms G Bennett	Lay Member	Yes	
Dr I Doull	Vice Chair & Consultant Respiratory Paediatrician	Yes	
Mrs A Dowden	Chair and Lay Member	No	
Dr N A Drage	Consultant Dental Radiologist	No	
Dr P H Edwards	GP	No	
Dr P Evans	Consultant Physician	Yes	
Mrs K Fisher	Lay Member	Yes	
Dr I J Kerby	Consultant Oncologist	Yes	
Mrs S J Kotecha	Research Dietician	No	
Mr P Lindsay	Consultant Obstetrician and Gynaecologist	Yes	
Dr A J Lipp	Nurse	Yes	
Mrs J Matthews	Pharmacist	Yes	
Mr J Owen	Lay Member	Yes	
Dr M D Page	Consultant Physician	No	
Dr R Price-Davies	Pharmacist	No	
Mrs S Warrell	Alternate Vice-Chair & Lay Member	Yes	

Also in attendance:

Name	Position (or reason for attending)
Mr C Phillips	Co-ordinator
